# Pharmacological modulation of GPR84 revealed by dual states structures and immune functional assays

**DOI:** 10.1038/s12276-026-01841-w

**Published:** 2026-09-10

**Authors:** Myung Kyung Choi, Dong Jin Park, Pankyung Kim, Hee Seong Choi, Sorin Myung, Youngki Yoo, Nienping Chang, Ga-Yeon Yoon, Hye Jin Kang, Sang-Jun Ha, Hyun-Soo Cho

**Affiliations:** 1 Department of Systems Biology, College of Life Science and Biotechnology, Yonsei University, Seoul, Republic of Korea; 2 Department of Biochemistry, College of Life Science and Biotechnology, Brain Korea 21 Project, Yonsei University, Seoul, Republic of Korea; 3 Department of Biotechnology, College of Life Science and Biotechnology, Yonsei University, Seoul, Republic of Korea; 4 Life Sciences Institute, University of Michigan, Ann Arbor, MI USA

**Keywords:** Monocytes and macrophages, Inflammatory diseases

## Abstract

G-protein-coupled receptor 84 (GPR84) is an orphan class A GPCR selectively activated by medium chain fatty acids and highly expressed in immune cells, where it modulates pro-inflammatory signaling. The structural basis of GPR84 inactivation and antagonism has remained unclear, limiting the rational design of pathway-selective modulators despite its clinical relevance in metabolic inflammation and fibrotic diseases. Here, we report cryo-electron microscopy structures of human GPR84 in inactive and active states. The 3.5 Å inactive structure bound to the antagonist GLPG1205 reveals a lid-like conformation of extracellular loop 2 and an inward reorientation of Arg172, with the antagonist head group blocking the allosteric sodium-binding site. Molecular dynamics simulations further support these findings, identifying an aberrant TM5, TM6 lateral entry gate. By contrast, the 3.17 Å agonist ZQ-16, Gαi complex, shows a rearranged toggle switch and comparative analyses highlight extracellular loop 2 conformational plasticity. Immune functional assays in THP-1 cells demonstrated that ZQ-16 elicited GPR84-dependent activation and cytokine production, which were effectively abrogated by GLPG1205. Mutagenesis combined with functional assays validates key ligand interactions, providing a framework for the rational design of pathway selective GPR84 modulators.

## Structures of GPR84 reveal mechanisms of immune modulation

G protein-coupled receptor 84 (GPR84) is a receptor primarily found in immune cells and is involved in metabolic and immune responses. This study addresses the challenge of understanding GPR84’s ligand specificity and signaling pathways, which are crucial for its role in diseases such as inflammation and cancer. Researchers used cryo-electron microscopy to capture the structures of GPR84 in both active and inactive states, bound to the agonist ZQ-16 and antagonist GLPG1205, respectively. These structures revealed how different ligands interact with GPR84, influencing its signaling pathways. The active state showed how ZQ-16 promotes pro-inflammatory responses, whereas the inactive state demonstrated how GLPG1205 inhibits these pathways. These findings provide a structural basis for designing selective modulators of GPR84, which could lead to new therapeutic strategies for diseases involving immune responses. Future research could explore endogenous ligands and diverse signaling mechanisms across cell types.

This summary was initially drafted using artificial intelligence, then revised and fact-checked by the author.

## Introduction

G protein-coupled receptor 84 (GPR84) is a class A GPCR predominantly expressed in immune cells such as monocytes, macrophages, and microglia^1–4^ and plays key roles in regulating metabolic and immune responses^5,6^. Unlike other human fatty-acid-sensing GPCRs, GPR40/120 (FFAR1 and FFAR4), which detect long chain fatty acids, and GPR41/43 (FFAR3 and FFAR2), which sense short chain fatty acids, GPR84 displays unique selectivity for medium chain fatty acids (MCFAs; C9–C14)^1,7^. Despite this specificity, its relatively low ligand affinity and the absence of a well-defined endogenous agonist leave it classified as an orphan receptor^3^.

GPR84 expression is strongly upregulated under inflammatory conditions, and its activation promotes macrophage polarization, cytokine release, and phagocytosis^1,6^. These properties link GPR84 to the pathophysiology of metabolic inflammation, fibrosis, and cancer^8–15^. Although canonical signaling proceeds through Gαi/o-mediated cAMP inhibition^1^, GPR84 can also engage β-arrestin, Gαq, and Gα12/13 in a ligand-dependent and cell-type-specific manner, suggesting that GPR84 possesses diverse downstream signaling capabilities^2,5^.

Functional studies have revealed both protective and pathogenic roles for GPR84. Loss-of-function models show impaired microglial activity in Alzheimer disease, hepatic lipid accumulation, atherosclerosis, abnormal eye development in *Xenopus laevis*, and regulation of murine skeletal muscle^16–21^. Conversely, GPR84 overexpression has been implicated in idiopathic pulmonary fibrosis (IPF), inflammatory bowel disease, ulcerative colitis, and neuropathic pain^22–24^. These bidirectional effects suggest that both agonists and antagonists may offer therapeutic value depending on disease context. However, this also highlights the potential for mechanism-based adverse effects, emphasizing the importance of aligning pharmacological strategies with disease-specific pathophysiology.

Pharmacological development has yielded selective small-molecule ligands that have deepened our understanding of GPR84 function. The synthetic agonist ZQ-16 (2-(hexylthio)-6-hydroxy-4(3H)-pyrimidinone) activates Gαi signaling and promotes pro-inflammatory macrophage responses^25^. By contrast, selective antagonist GLPG1205 (9-(2-cyclopropylethynyl)-2-[[(2S)-1,4-dioxan-2-yl]methoxy]-6,7-dihydropyrimido[6,1-a]isoquinolin-4-one), developed by Galapagos NV, exhibits antifibrotic activity but failed to meet primary end points in a phase II IPF trial^26–30^. Notably, GLPG1205’s defined scaffold provides a valuable chemical probe for structural studies. This underscores the need for high-resolution structural information particularly in the inactive state to enable rational design of pathway-selective modulators.

Here, we report cryo-electron microscopy (cryo-EM) structures of human GPR84 in dual conformations. The 3.17 Å active-state structure bound to selective agonist ZQ-16 and Gαiβγ heterotrimer captures the receptor in a fully active conformation under near-physiological conditions. We also report that the first inactive-state structure of GPR84 bound to GLPG1205 reveals the molecular basis for antagonist binding and receptor inactivation resolved at 3.5 Å. These structures offer key insights into ligand recognition and activation mechanisms, laying a structural bias for the rational design of GPR84 targeted therapeutics. These studies identified key ligand-contacting residues and demonstrated that distinct ligands drive specific signaling outcomes in macrophages, highlighting the biological relevance of ligand-specific GPR84 activation. Together, these findings define the molecular determinants of ligand recognition and signaling specificity, providing a structural framework to guide the rational design of selective modulators and to explore the receptor’s diverse physiological roles in disease contexts.

## Methods

### Construct design and protein expression

The human GPR84 gene (UniProt ID: Q9NQS5, residues 4–390) was codon-optimized and synthesized by GenScript. For structural stabilization in the inactive conformation, a thermostabilized apocytochrome b562RIL (BRIL) was inserted into the third intracellular loop (ICL3, residue 212–309, termed GPR84-BRIL). The heptapeptide sequence ERARSTL, derived from the sixth helix of the human adenosine A2A receptor (A2AR), was inserted between the C terminus of BRIL and GPR84 residue 309 to maintain helical continuity between TM helices 5 and 6. A tobacco etch virus (TEV) protease cleavage site followed by a 10× His tag was appended to the C terminus of GPR84 for affinity purification and subsequent tag removal. For capturing GPR84 in its active conformation, the codon-optimized human GPR84 gene (residue 4–390) sequence was cloned into the pFastBac1 vector as a fusion with N-terminal signal peptide GP67-Flag tag (DYKDDDDK) and C-terminal TEV protease cleavage site between 10× His tag. Gαi1 was cloned into pFastBac1 vector, and 6× His-tagged human Gβ1 and human Gγ2 were cloned into pFastBac dual vector. The GPR84 and G protein baculovirus were generated and amplified to high titer P3 (>108 viral particles per ml). The expression in *Spodoptera frugiperda* (Sf9) cells (2 × 10^6^ cells per ml, ESF921 medium, Expression Systems) infected with GPR84-BRIL virus at a multiplicity of infection (MOI) of 5 for inactive structure and co-infected with GPR84-Gαi-Gβγ in a 1:2:1 ratio at an MOI of 5 for active structure. Cells were harvested by centrifugation at 72 h post-infection, washed in phosphate-buffered saline (PBS) buffer, and stored at −80 °C until use.

### Purification of inactive-state GPR84–BRIL complex

Cell pellets were resuspended in 25 mM Tris–HCl pH 7.5, 300 mM NaCl containing a protease inhibitor (PI) cocktail (Complete™, Roche) and lysed by Dounce homogenization. Following ultracentrifugation at 100,000×*g* for 1 h at 4 °C, the membrane pellets were resuspended in same buffer. To remove peripheral and loosely associated proteins, membranes were sequentially washed with buffers containing 1 M, 500 mM, and 300 mM NaCl, respectively, followed by homogenization and centrifugation at 100,000×*g* for 30 min at 4 °C after each wash. The washed membranes were solubilized for 2 h at 4 °C in solubilization buffer containing 25 mM Tris–HCl pH 7.5, 300 mM NaCl, 1.5% (w/v) *n*-dodecyl-β-D-maltopyranoside (DDM), 0.3% (w/v) cholesterol hemisuccinate (CHS), and 15 μM GLPG1205 (Galapagos NV, Mechelen, Belgium). Insoluble debris was removed by ultracentrifugation at 100,000×*g* for 45 min at 4 °C. The clarified supernatant was incubated with TALON metal affinity resin (TaKaRa) at 4 °C for 2 h. The resin was washed with buffer containing 25 mM Tris–HCl pH 7.5, 300 mM NaCl, 10 mM imidazole, 0.05% (w/v) DDM, 0.005% (w/v) CHS, and 3 μM of the GLPG1205. Bound protein was eluted with buffer containing 300 mM imidazole, 0.03% (w/v) DDM, 0.006% (w/v) CHS, and 3 μM GLPG1205. The eluted protein was concentrated using a 100 kDa cut-off centrifugal concentrator (Amicon Ultra, Millipore) and desalted by buffer exchange into 25 mM Tris–HCl pH 7.5, 300 mM NaCl, 0.03% (w/v) DDM, 0.006% (w/v) CHS, and 1 μM GLPG1205 using a PD Midi trap G-25 desalting column (Cytiva) to remove imidazole. The desalted protein was incubated with TEV protease at a mass ratio of 1:20 (TEV:protein) overnight at 4 °C to remove the C-terminal His tag. The sample was then subjected to a second round of TALON affinity purification of the cleaved target protein from uncleaved protein. The flow through containing tag-free GPR84 was collected, concentrated, and further purified by size exclusion chromatography (SEC) on a Superdex 200 Increase 10/300 GL column (Cytiva) equilibrated in SEC buffer containing 25 mM Tris–HCl pH 7.5, 300 mM NaCl, 0.0 3% (w/v) DDM, 0.006% (w/v) CHS, and 1 μM GLPG1205. Peak fractions were collected and used for subsequent experiments. The anti-BRIL Fab and anti-Fab nanobodies (Nbs) were expressed in *Escherichia coli* and purified as previously reported^31^. The purified receptor was incubated with 1.5-fold and 2-fold molar excess of anti-BRIL Fab Nb and anti-Fab Nb, at 4 °C for 1 h. The mixture was concentrated to 500 µl and injected into an SEC column Superdex 200 10/300 GL equilibrated with the SEC buffer containing 25 mM Tris–HCl pH 7.5, 300 mM NaCl, 0.01% (w/v), LMNG 0.001% (w/v) CHS, and 1 μM GLPG1205. The peak fractions were concentrated to 12 mg/ml for cryo-EM studies.

### Purification of active-state GPR84–Gαi protein complex

Cell pellets were resuspended in 50 mM HEPES pH 7.5, 150 mM NaCl, 3 mM MgCl_2_ containing a PI (Complete, Roche). The suspension was further supplemented with 25 mU/ml Apyrase (NEB) before dounce homogenization. The membranes were solubilized by adding 1.5% (w/v) DDM (Anatrace), 0.15% (w/v) CHS (Anatrace), 13.5 μM ZQ-16 (Sigma-Aldrich) and rotating at 4 °C for 2 h. The solubilized fraction was isolated by ultracentrifugation at 100,000×*g* at 4 °C for 40 min and incubated at 4 °C for 2 h with TALON metal affinity resin (TaKaRa). The resin was washed with five column volumes of washing buffer containing 50 mM HEPES (pH 7.5), 150 mM NaCl, 3 mM MgCl_2_, 10 mM imidazole, 0.05% (w/v) DDM, 0.005% (w/v) CHS, and 2.7 μM of ZQ-16. The complex protein was then eluted with elution buffer containing 50 mM HEPES (pH 7.5), 150 mM NaCl, 3 mM MgCl_2_, 300 mM imidazole, 0.05% (w/v) DDM, 0.005% (w/v) CHS, and 2.7 μM of ZQ-16 giving 3 min incubation for each elution. Elution sample was batch-bound to equilibrated M2 anti-Flag affinity resin, rotating at 4 °C for 1 h. The resin was packed into a glass column and washed with five column volumes of 20 mM HEPES (pH 7.5), 150 mM NaCl, 3 mM MgCl_2_, 0.03% (w/v) DDM, 0.003% (w/v) CHS, and 2.7 μM of ZQ-16 before elution in the same buffer with 0.5 mg/ml Flag peptide also giving 3 min incubation for each elution. GPR84 complexes were then concentrated using a Vivaspin 20 centrifuge concentrator (Sigma-Aldrich) with a molecular cut-off of 100 kDa and further purified by SEC on Superose 6 increase 10/300 GL gel filtration column (Cytiva) with SEC buffer containing 20 mM HEPES pH 7.5, 150 mM NaCl, 3 mM MgCl_2_, 0.03% (w/v) DDM, 0.003% (w/v) CHS, and 1.35 μM ZQ-16. Peak fractions were concentrated to 3 mg/ml for cryo-EM studies.

### Cryo-EM grid preparation and data collection

The inactive-state, GPR84-BRIL, Fab, Nb, and GLPG1205 complex sample (4 µl) was applied to negatively glow-discharged holey carbon 300-mesh grids (Quantifoil R1.2/1.3) and flash-frozen in liquid ethane using a Vitrobot Mark IV (FEI) operated at 100% humidity and 4 °C (blot time: 4 s; blot force: 0; wait time: 5 s). Cryo-EM data were collected on a Titan Krios G4 transmission electron microscope (Thermo Fisher Scientific) operated at 300 kV and equipped with a Gatan K3 BioQuantum detector in electron-counting mode. Micrographs were acquired at a nominal magnification of 130,000×, corresponding to a calibrated pixel size of 0.664 Å per pixel. A total of 8,575 movies were collected, each comprising 48 frames over a 3.85 s exposure, with a total dose of 69 electrons per Å^2^ and defocus values ranging from −0.7 μm to −2.0 μm.

For the active-state structure, 4 μl of the purified GPR84–ZQ-16–Gαiβγ complex was applied to glow-discharged Quantifoil R1.2/1.3 300-mesh grids and vitrified under the same conditions (blot time: 4 s; blot force: 0; wait time: 0 s). Data collection was performed on a Titan Krios microscope (Thermo Fisher Scientific) operated at 300 kV and equipped with a Falcon IV direct electron detector in electron-counting mode. Images were recorded at a nominal magnification of 160,000×, yielding a calibrated pixel size of 0.7052 Å per pixel. A total of 16,820 movies were acquired with a total dose of 50 electrons per Å^2^ and a defocus range of −0.8 μm to −2.1 μm.

### Cryo-EM image processing

Motion correction and contrast transfer function (CTF) estimation were performed using the Patch motion correction and Patch CTF algorithms in cryoSPARC (v4.2.1 for the GPR84-BRIL-Fab-Nb-GLPG1205 complex and v4.0.0 for the ZQ-16–GPR84–Gαiβγ complex)^32^. For the inactive complex, particles were initially picked using the blob picker, followed by multiple rounds of 2D classification to remove poorly defined particles. Ab initio reconstruction and non-uniform refinement resulted in a final map at a global resolution of 3.5 Å, as determined by the Fourier shell correlation (FSC) = 0.143 cut-off. For the active complex, particles were picked using the same blob picker algorithm, and after 2D classification, particles were selected for subsequent 3D analysis. Ab initio modeling and non-uniform refinement yielded a final reconstruction at a resolution of 3.17 Å, based on the FSC = 0.143 cut-off.

### Model building and refinement

The AlphaFold3 (ref. ^33^) predicted that structures of GPR84-BRIL and BRIL-Fab-Nb (PDB: 6WW2) were used as the initial model for model rebuilding and refinement against the cryo-EM density map. The model was first docked into the map using UCSF Chimera^34^, followed by iterative manual adjustment and rebuilding in COOT. Real-space refinement was carried out using PHENIX software^35^. For the GPR84–Gαiβγ complex, the initial model (PDB: 8G05 and 8J19) was rigid body fitted into the cryo-EM density using Chimera and further refined in PHENIX. Geometric restraints for the ligands GLPG1205 and ZQ-16 were generated using the eLBOW module in PHENIX. Ligands were fitted based on clearly defined density features, and their stereochemistry was validated during refinement. The quality of the final models was assessed using the comprehensive model validation tools and MolProbity in PHENIX. Structural figures were generated using UCSF Chimera and PyMOL^36^. Model building and refinement statistics are summarized in Supplementary Table 1.

### Bioluminescence resonance energy transfer (BRET) assay

HEK293 cells were cultured in complete Dulbecco’s modified Eagle's medium (DMEM) supplemented with 10% fetal bovine serum (FBS), 1% penicillin G, and 100 μg/ml streptomycin, maintained at 37 °C in an atmosphere containing 5% CO_2_. On day 1, cells were seeded into 6-well plates with white walls and clear bottoms at a density ranging from 350,000 to 400,000 cells per well in complete media. Before transfection, a DNA mixture was prepared by combining receptor plasmid and mini Gαi, β-arrestin1 (β-arr1), or β-arrestin2 (β-arr2) plasmids at a 1:6 molar ratio in a total volume of 250 μl Opti-MEM Reduced Serum Medium per well. Then, 2.1 μl of Transit 2020 transfection reagent was added, and the mixture was incubated at room temperature for 30 min. The transfection complex was gently added to the plated HEK293 cells, which were subsequently incubated overnight at 37 °C with 5% CO_2_. The following day, cells were detached and reseeded into 96-well plates precoated with poly-L-lysine, at 200 μl per well in DMEM containing 1% dialyzed FBS, 1% penicillin G, and 100 μg/ml streptomycin. After overnight incubation, the medium was removed and the cells were washed once with 60 μl of assay buffer (1× HBSS, 20 mM HEPES, pH 7.4). For agonist assay, 30 μl of assay buffer (with 0.1% bovine serum albumin (BSA)) was added to each well. Then, 30 μl of a 3× drug in assay buffer was added and incubated for 10 min at room temperature. Afterward, 30 μl of 3× substrate coelenterazine h (final 5 μM) prepared in assay was added to each well and allowed to incubate for 10 min. For the antagonist assay, after addition of 30 μl assay buffer with 0.1% BSA, 30 μl of a 4× antagonist solution (GLPG1205 in assay buffer) was added and incubated for 10 min at room temperature. Afterward, 30 μl of 4× EC80 of agonist solution (ZQ16 in assay buffer) was added and incubated for 10 min. Finally, 30 μl of 4× substrate was added to each well and allowed to incubate before measurement. BRET was measured at emission wavelengths 480 nm and 540 nm using a Tristar 5 plate reader, with six successive scans performed. The resulting data were processed and analyzed with GraphPad Prism 10 software.

### Cell culture and macrophage polarization

Human THP-1 cells (ATCC, TIB-202) were cultured in RPMI-1640 medium (Biowest) supplemented with 10% FBS (Gibco, cat. no. 16000-044) and 1% penicillin–streptomycin (Gibco). For macrophage differentiation, 2.5 × 10^5^ cells were seeded in 12-well tissue-culture plate in 1 ml medium and cultured in RPMI-1640 containing 10% heat-inactivated FBS, 1% penicillin–streptomycin, and 12.5 ng/ml phorbol 12-myristate 13-acetate (PMA; Sigma-Aldrich). After 24 h of PMA stimulation, the medium was replaced, and cells were allowed to rest for 24 h without any stimulation to promote differentiation. Cells were subsequently stimulated with 100 ng/ml lipopolysaccharide (LPS, InvivoGen) by medium replacement. GPR84 agonist and antagonist were performed for 16 h of LPS stimulation. The GPR84 antagonist GLPG1205 was added 15 min before medium replacement with fresh medium containing both ZQ-16 and GLPG1205. For each treatment, the existing medium was replaced with fresh medium to avoid residual stimulation.

### Staining for flow cytometry analysis and cytokine level assessment

Adherent cells were detached from tissue-culture plate using a cell scraper, washed with PBS containing 2% FBS and incubated with human Fc block (BD Bioscience, Cat# 564220) for 20 min at 4 °C. Subsequently, cells were stained with antibodies against extracellular markers for 20 minutes at 4 °C in the presence of LIVE/DEAD kit (Invitrogen) to exclude dead cells from analysis. The following antibodies were used: CD11b (ICRF44), CD80 (2D10), CD86 (IT2.2), and PD-L1 (29E.2A3) (all from BioLegend, clones indicated in parentheses), CD14 (MφP9, BD Biosciences), and GPR84 (AGR-052-F, Alomone Labs, LTD). Data were acquired on a CytoFLEX LX (Beckman Coulter) and analyzed using FlowJo software. For cytokine level assessment process, culture supernatant was used. Following the manufacturer’s protocol, levels of five cytokines (IL-1β, CCL2, CCL3, CXCL8, and CXCL10) were measured using the LEGENDplex multiplex system (BioLegend). Data were acquired on a CytoFLEX (Beckman Coulter) and analyzed with the software provided by the manufacturer.

### Molecular dynamics simulations

Molecular dynamics (MD) simulations were conducted for human GPR84 in complex with the antagonist GLPG1205 (PDB: 9V6T, GPR84–bRIL–GLPG1205 complex). The BRIL fusion protein inserted into the ICL3 was removed, and 9 a, a linker corresponding to the native GPR84 sequence, was introduced to restore loop connectivity^8^. To construct the simulation system, the protein ligand complex was embedded in a 1-palmitoyl-2-oleoyl-sn-glycero-3-phosphocholine (POPC) bilayer within a 105 × 105 × 104 Å³ simulation box using CHARMM-GUI^37^. The system was solvated with 27,729 TIP3P water molecules, and 150 mM NaCl was added to neutralize the system and approximate physiological ionic strength. The final composition included 288 POPC molecules and the protein–ligand complex. MD simulations were performed using GROMACS 2025.1 (ref. ^38^) with the CHARMM36 (refs. ^39–41^) force field applied to proteins, lipids, and ions. The ligand topology was generated using the CHARMM General Force Field (CGenFF)^42^. Following energy minimization with the steepest descent algorithm, the system was equilibrated in the NVT (number of particles, volume and temperature) ensemble for 2 ns to allow relaxation of solvent and lipid components, followed by NPT (number of particles, pressure and temperature) equilibration in which positional restraints were progressively released in stages from lipids and solvent, protein side chains, the ligand, and finally the protein backbone (2.5 ns per stage). After equilibration, production simulations were carried out for 2 μs with a 2 fs time step. Periodic boundary conditions were applied throughout, and the system was maintained at 310 K and 1 bar. Long-range electrostatic interactions were treated with the particle mesh Ewald^43^ method using a 12 Å cut-off, and van der Waals interactions were smoothly switched to zero between 10 Å and 12 Å. All bonds involving hydrogen atoms were constrained with the LINCS algorithm^44^, enabling the 2 fs integration time step. Each system was simulated in triplicate with different initial velocities. Trajectory and structural data collected during the simulations were analyzed using VMD^45^.

### Cell surface expression assay

Cell surface expression of wild-type (WT) and mutant GPR84 constructs was assessed using an ELISA-based chemiluminescence assay. HEK293 cells were transiently transfected with plasmids encoding FLAG-tagged receptor variants using Lipofectamine 2000 (Thermo Fisher Scientific) according to the manufacturer’s protocol. After 48 h post-transfection, cells were seeded into 384-well white plates (20,000 cells per well). Cells were fixed with 20 μl per well of 4% paraformaldehyde in PBS for 10 min at room temperature and washed twice with 40 μl per well of PBS. Nonspecific binding was blocked by incubation with 20 μl per well of 5% BSA in PBS for 1 h at room temperature. The wells were then incubated with 20 μl per well of HRP-conjugated mouse monoclonal anti-FLAG M2 antibody (Sigma-Aldrich, Cat. no. A8592), diluted 1:10,000 in 5% BSA/PBS, for 1 h at room temperature. After five washes with 80 μl per well of PBS, 20 μl per well of SuperSignal™ ELISA Pico Chemiluminescent Substrate (Thermo Fisher Scientific) was added. The luminescence signal was measured immediately at 425 nm using a CLARIOstar Plus multilabel plate reader (BMG LABTECH) within 5 min after substrate addition. To assess relative surface expression, signal intensities from each mutant were normalized to those from WT GPR84, which was included on every assay plate. All data were processed and analyzed using GraphPad Prism 9 software. For selected mutants exhibiting significantly reduced surface expression compared with WT (*P* < 0.05), transfection conditions were adjusted by increasing the amount of plasmid DNA (200–300 ng per well) to achieve more comparable expression levels before functional assays.

## Results

### Overall structure of the GLPG1205–GPR84 complex

**Fig. 1:**
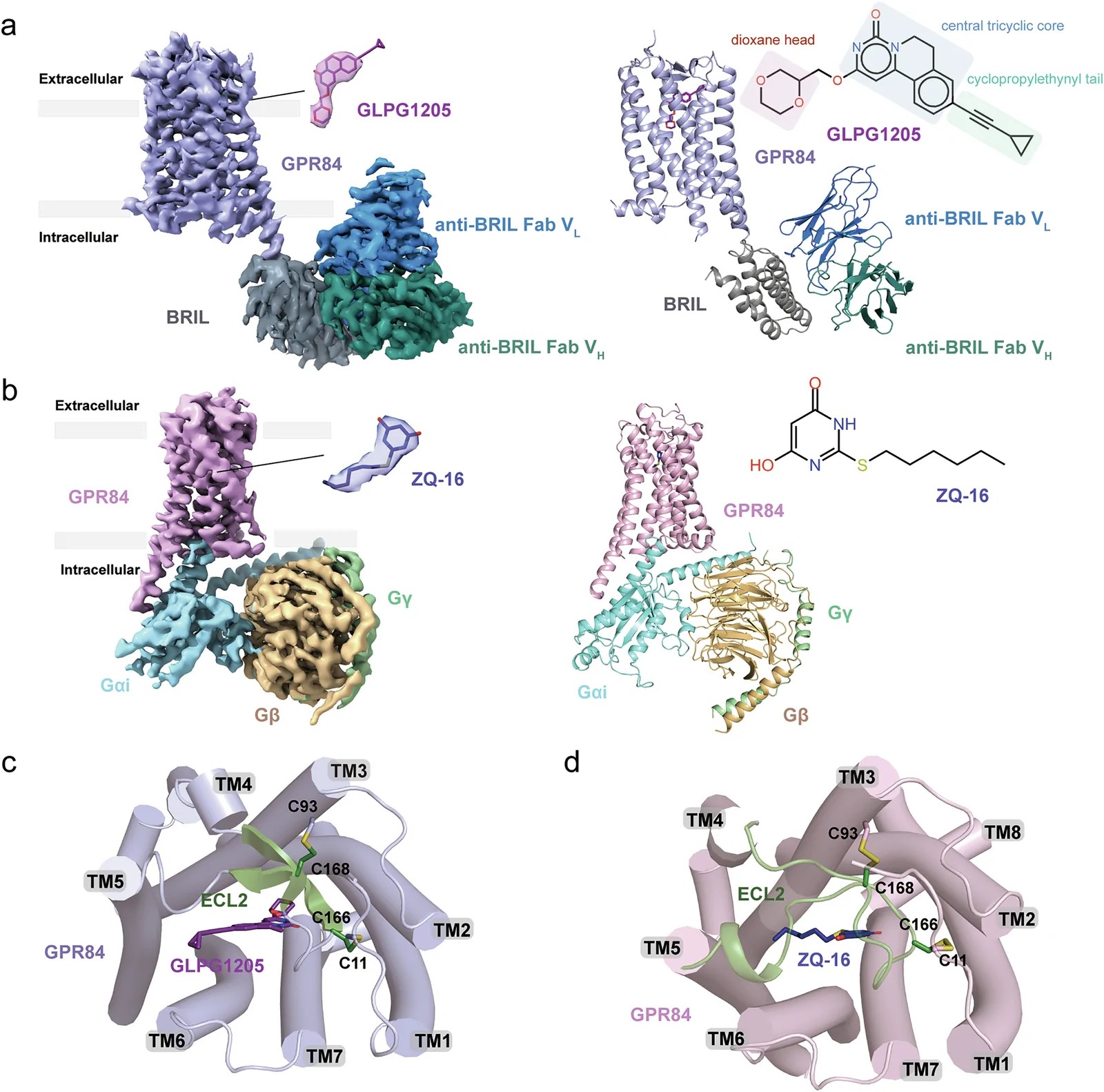
Cryo-EM structures of human GPR84 in inactive and active states. **a** Cryo-electron microscopy (EM) structure of the GLPG1205–GPR84–BRIL–Fab complex. Overall map (left), model of GPR84 (light blue) with BRIL (gray) and Fab fragments (blue and green) (right) and chemical structure of GLPG1205 (purple). **b** Cryo-EM structure of the ZQ-16–GPR84-Gαi complex. Overall map (left), model of GPR84 (light pink), Gαi (cyan), Gβ (orange), and Gγ (light green) (right) and chemical structure of ZQ-16 (dark blue). **c** Inactive structure of GPR84 highlighting the ECL2 (green) lid conformation stabilized by dual disulfide bonds. **d** Active structure of GPR84 showing ECL2 (green) loop and disulfide connectivity. ECL, extracellular loop; TM, transmembrane.

To obtain a stable inactive conformation of GPR84, we explored multiple construct engineering strategies and extensive biochemical screening. We adopted a BRIL fusion strategy, systematically evaluating insertion sites within ICL3 as fiducial marker. Incorporation of a short helix segment (ERARSTLQK) derived from the A2A adenosine receptor (A2aAR) downstream of BRIL markedly enhanced protein stability^31,46^. We purified the BRIL-fused GPR84 and incubated it with an anti-BRIL Fab and anti-Fab Nbs^46^, which served as fiducial markers (Supplementary Fig. 1a,d). Using this construct, we determined the cryo-EM structure of human GPR84 in complex with the antagonist GLPG1205 at an overall resolution of 3.5 Å (Fig. 1a and Supplementary Fig. 1c,e). Electron density maps showed that the Fab and Nb adopt a more membrane proximal orientation (Supplementary Fig. 1d), likely due to a kink at the BRIL–TM5 junction. To further improve the resolution of the receptor, the constant regions of Fab and Nb were masked out during local refinement, thereby enhancing map quality in the transmembrane (TM) domain (Supplementary Fig. 1b).

The final model includes residues N terminus Asn8 (Asn8^NT^)–Leu385^8.58^ (Ballesteros–Weinstein numbering in superscript^47^) of GPR84 and adopts the canonical class A GPCR fold comprising 7TM (Supplementary Fig. 1f). A BRIL domain (residues Ala1–Gly107, chain B) was clearly resolved in the density together with the variable region of the bound Fab. Although the linker between TM6 and BRIL was disordered, BRIL formed a continuous helix with TM5 (Fig. 1a). Consistent with this, BRET-based functional assays confirmed that GLPG1205 completely abolishes GPR84-mediated Gαi signaling, with an EC_50_ of 0.163 nM, supporting the assignment of this conformation as an inactive state (Supplementary Figs. 3b and 10a and Supplementary Table 2).

Inactive GPR84 structure has the lid-like conformation of the second extracellular loop (ECL2), which adopts a β-strand stabilized fold that occludes the orthosteric-binding pocket (Fig. 1c and Supplementary Fig. 3a). This configuration is stabilized by two disulfide bonds: between TM3 Cys93^3.25^ and Cys168 of ECL2 (Cys168^ECL2^), Cys11^NT^ and Cys166^ECL2^ (Fig. 1c). Importantly, BRET assays demonstrated that Cys166 ^ECL2^A and Cys168^ECL2^A mutations within ECL2 have a key role in maintaining structural stabilization of GPR84 (Supplementary Figs. 3b and 10a and Supplementary Table 2). Specifically, Cys166^ECL2^A exhibited a partial loss, whereas Cys168^ECL2^A resulted in a loss-of-function phenotype.

### Overall structure of the ZQ-16–GPR84–Gαiβγ complex and ligand binding site

To elucidate the activation mechanism of GPR84, we determined the cryo-EM structure of GPR84 in complex with the agonist ZQ-16 and Gαiβγ at a resolution of 3.17 Å (Fig. 1b and Supplementary Fig. 2a,c,e). Cryo-EM density enabled model building of GPR84 from Ser10^NT^ to Ala380^8.53^, including that the ligand-binding pocket (Supplementary Fig. 2f) except ICL3 (Arg218^ICL3^–Ser312^6.28^) was disordered. Notably, TM5 extends beyond the typical helical length observed in class A GPCRs. Within the G protein heterotrimer, the β subunit is well resolved, whereas the γ subunit and the α-helical domain of Gαi are partially disordered (Supplementary Fig. 2b,d).

**Fig. 2:**
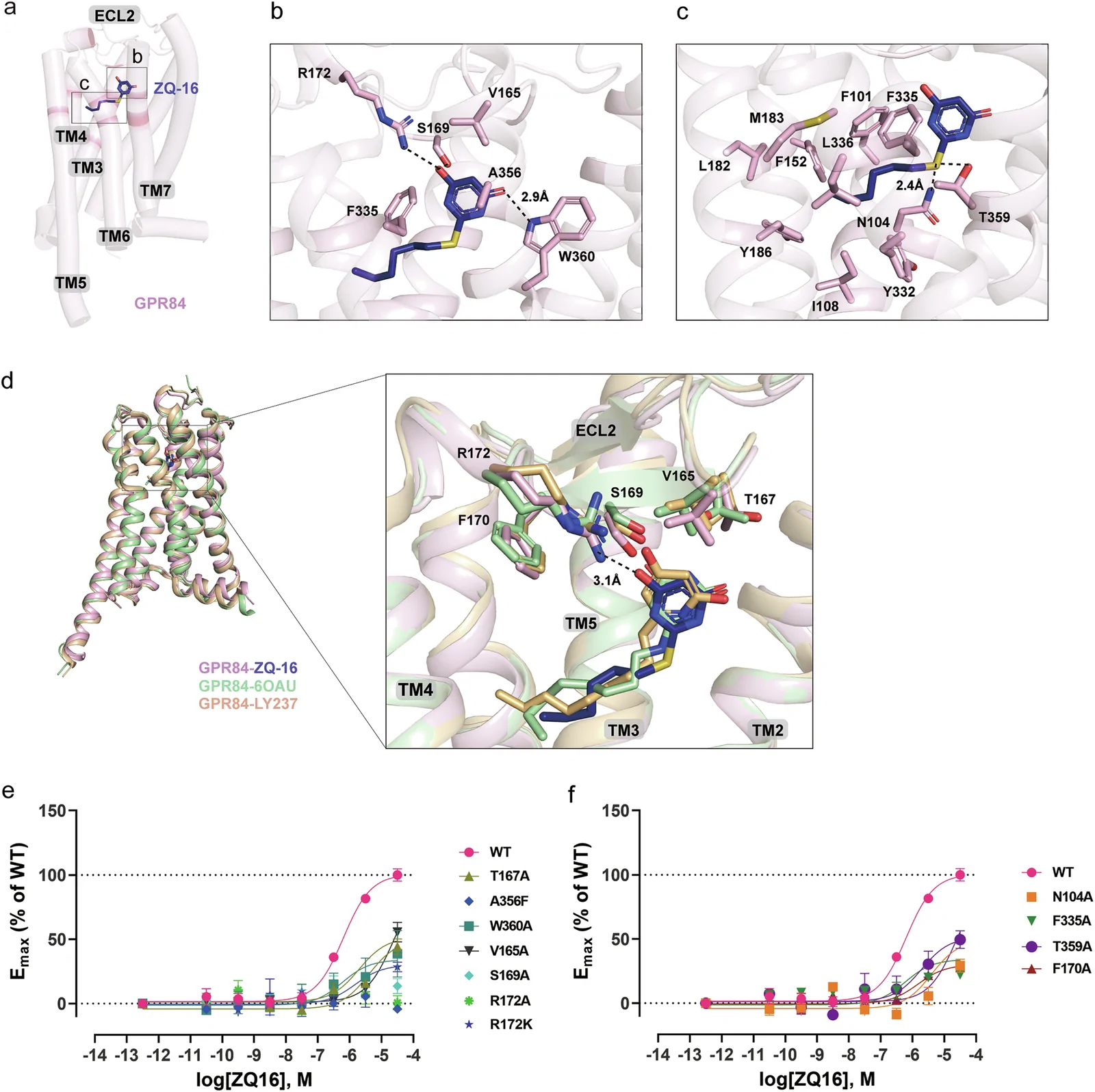
Binding mode of the agonist ZQ-16 in GPR84. **a** Side view of ZQ-16 (dark blue) bound within the orthosteric pocket of GPR84 (pink), showing interaction residues highlighted (magenta). **b** Polar interactions of ZQ-16 head group and hydrogen bonds. **c** Hydrophobic and aromatic interactions with ZQ-16 and hydrogen bonds. **d** Structural comparison of ZQ-16 (dark blue)-bound GPR84 (light pink) with other agonist-bound structures (6OAU, PDB: 8G05, light green; LY237, PDB: 8J19, light orange). **e**, **f** Functional validation of binding determinants by the bioluminescence resonance energy transfer assay. Mutations at key residues of GPR84 markedly reduced ZQ-16-induced activation compared with WT. Data are mean ± sem of *n*  =  3 biological replicates. Dose–response curves were fitted using nonlinear regression. Statistical significance was assessed using one-way analysis of variance. Hydrogen bonds are represented by black dashed lines. ECL, extracellular loop; *E*_max_, maximum effect; WT, wild-type.

In GPR84 structure, ZQ-16 occupies the orthosteric pocket in an amphipathic configuration (Fig. 2a and Supplementary Fig. 4b). Its hydrophobic hexyl tail interacts with the hydrophobic cleft formed by TM3 to TM6, whereas the polar pyrimidine head group engages in hydrogen bonding with ECL2 and TM7 (Fig. 2a, b). Notably, the ECL2 adopts a lid-like conformation in previous GPR84 structures and our inactive structure^8,48^. In the ZQ-16 bound structure, ECL2 adopts a disordered loop configuration instead of the β sheet conformation (Fig. 1d). Structural rearrangement appears to be driven by the slightly deeper insertion of ZQ-16 into the orthosteric pocket and interacting ECL2 residues repositioned (Fig. 2d). The Phe170^ECL2^, which forms a cation–π interaction with Arg172^ECL2^, is also displaced downward, further contributing to the disruption of the β sheet architecture. The conformational flexibility of ECL2 is consistent with a previously proposed MD simulations^8^.

The 4,6-hydroxyl group of pyrimidine ring in ZQ-16 forms hydrogen bonds with polar residues Arg172^ECL2^, Ser169^ECL2^, and Trp360^7.43^ and alanine substitutions of these residues substantially impaired the efficacy of ZQ-16 (Fig. 2b, e). Consistent with this interaction network, Ser169 ^ECL2^A and Arg172 ^ECL2^A resulted in an inactive phenotype, whereas Arg172 ^ECL2^K led to a loss-of-function phenotype. The pyrimidine ring is also in proximity with Val165^ECL2^, Thr167^ECL2^, Ala356^7.39^, Leu100^3.32^, and Phe335^6.51^, supporting a network of van der Waals interactions (Fig. 2e, f). In line with this, Val165^ECL2^A showed reduced potency, whereas mutations at Thr167^ECL2^ and Phe335^6.51^A resulted in a loss-of-function phenotype. In addition, substitution of Ala356^7.39^ with bulkier or charged residues (Ala356^7.39^F and Ala356^7.39^D) rendered the receptor inactive, indicating that steric and chemical compatibility at this position are critical for ligand accommodation. The thioether linker of ZQ-16 lies in close proximity to Leu100^3.32^, enabling hydrophobic interactions (Supplementary Fig. 4e) and hydrogen bond with Asn104^3.36^ and Thr359^7.42^ (Fig. 2b). Consistently, Asn104^3.36^A resulted in an inactive phenotype, whereas Thr359^7.42^A exhibited a partial loss-of-function phenotype (Fig. 2f). Moreover, Trp360^7.43^A and Phe170^ECL2^A mutations led to loss-of-function phenotypes.

Structural alignment of previously reported active-state GPR84 structures 6OAU (PDB: 8G05, 6-*n*-octylaminouracil)^8^ and LY237 (PDB: 8J19, 6-nonylpyridine-2,4-diol)^48^ and TM domains reveal minimal conformational deviation, with Cα RMSD of 0.56 Å and 0.71 Å (Supplementary Fig. 4a,c and Supplementary Fig. 13). Despite the high degree of structural conservation, notable differences were observed in the ECL2 and the ligand binding site (Fig. 1d and Supplementary Fig. 4d–f). Despite being two carbon shorter than the tail of 6OAU, the thioether moiety in ZQ-16 imparts conformational flexibility, enabling it to occupy a similar hydrophobic subpocket (Supplementary Fig. 4a,e). Key contacts include Phe101^3.33^, Tyr332^6.48^, Ile108^3.40^, Tyr186^5.46^, Phe152^4.57^, Leu336^6.52^, and Phe335^6.51^, forming a tightly packed, nonpolar and hydrophobic environment (Fig. 2c). Although Leu182^5.42^ and Met183^5.43^ are positioned at a relatively longer distance, they contribute to form hydrophobic pocket. GPR84 displays marked sensitivity to the length of the tail of the ligand, with optimal activation observed for 9–12 carbons^1,2,7^. ZQ-16 structure also supports that this length selectivity arises from a hydrophobic blocking wall formed by Phe152^4.57^, Leu182^5.42^, and Tyr186^5.46^ on TM4,5 (Supplementary Fig. 4e), which limits the depth of tail insertion^48^. Mutations of most ZQ-16 interacting residues and the blocking-wall residues markedly reduced GPR84 activity (Fig. 2e, Supplementary Figs. 4g,h and 10a, and Supplementary Table 2) in BRET assay. Consistent with their roles, Leu182^5.42^A, Tyr186^5.46^A, and Met183^5.43^A exhibited reduced potency, whereas Phe152^4.57^A showed increased potency. In addition, Tyr69^2.53^A and Phe101^3.33^A resulted in partial loss, whereas Tyr332^6.48^A, Ile108^3.40^A, and Leu100^3.32^A mutations led to inactive or loss of function phenotype.

### Antagonist GLPG1205 ligand-binding site

**Fig. 3:**
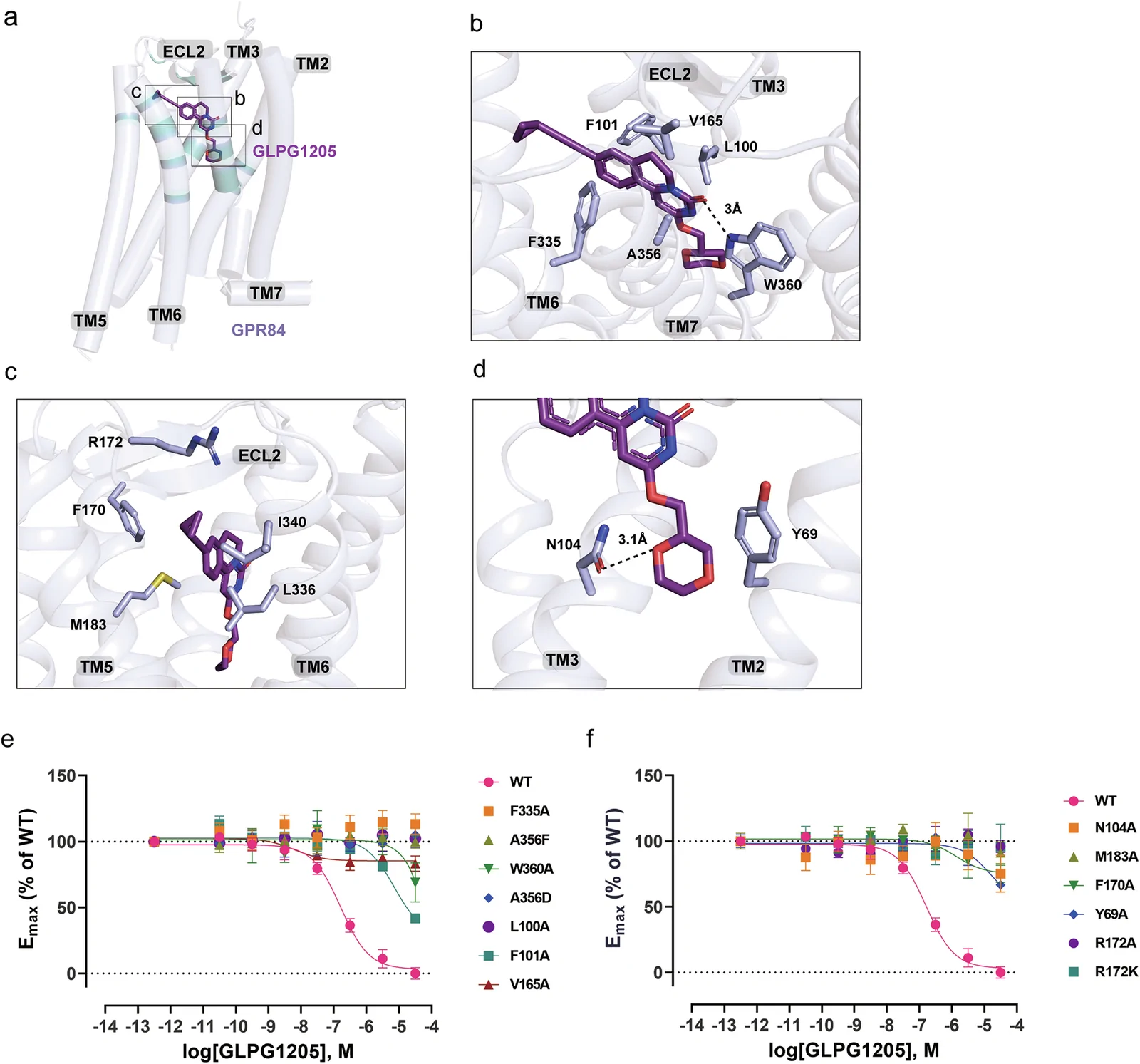
Binding mode of the antagonist GLPG1205 in GPR84. **a** Side view of GLPG1205 (purple) bound in the orthosteric pocket of GPR84 (light blue), with interaction residues highlighted (deep teal).** b** Hydrophobic and polar interactions of GLPG1205 and hydrogen bond. **c** Interaction residues with GLPG1205 cyclopropylethynyl tail within the binding cavity. **d** Hydrogen bond formed by the dioxane head group of GLPG1205 at the TM2–TM3 interface. **e**, **f** Mutational analysis of residues in the GPR84-binding pocket, showing that alanine or substitution mutations markedly impaired GLPG1205-induced responses in bioluminescence resonance energy transfer assays. Data are mean ± sem of *n*  =  3 biological replicates. Dose–response curves were fitted using nonlinear regression. Statistical significance was assessed using one-way analysis of variance. Hydrogen bonds are represented by black dashed lines. ECL, extracellular loop; *E*_max_, maximum effect; TM, transmembrane; WT, wild-type.

Structurally, GLPG1205 consists of three distinct moieties: a central tricyclic core (a rigid, fused three-ring core), a dioxane head group, and a cyclopropylethynyl tail (Fig. 1a). The cryo-EM density of the GLPG1205–GPR84 complex allowed precise modeling of the central scaffold and dioxane ring. Although the density for the tail was relatively weak, its rigid, non-rotatable linkage to the central core enabled confident placement of its conformation. The ligand adopts a deeply and broadly embedded pose, spanning TM2, TM3, TM5, TM6, TM7, and ECL2, and engages an extensive network of interactions (Fig. 3a). Notably, GLPG1205 exhibits dioxane moiety inserted toward the intracellular region, whereas the tail projects toward the extracellular surface in different direction observed in agonists. The binding orientation of GLPG1205 was also confirmed through replication of MD simulations (Supplementary Fig. 6a–c).

The central tricyclic core is embedded within a cavity formed by TM2, TM3, TM6, and TM7 (Fig. 3b). The rigid scaffold forms stabilizing hydrophobic contacts with Leu100^3.32^, Val165^ECL2^, and Ala356^7.39^, while being sandwiched between Phe101^3.33^ and Phe335^6.51^ through aromatic-stacking interaction (Fig. 3c). These structural features are supported by BRET assays, as mutations Phe335^6.51^A, Ala356^7.39^F, Ala356^7.39^D, Leu100^3.32^A, Phe101^3.33^A, and Val165^ECL2^A impaired GLPG1205-mediated inhibition of GPR84 signaling (Fig. 3e). Consistent with their roles in stabilizing the antagonist, substitutions at these positions produced loss-of-function phenotypes, whereas Phe101^3.33^A caused only a partial loss of function. Notably, both Ala356F and Ala356D yielded inactive phenotypes. A key hydrogen bond is formed between the indole NH of Trp360^7.43^ and the lactam carbonyl of the central scaffold, anchoring its orientation, and the Trp360^7.43^A mutation diminished GLPG1205-mediated GPR84 inhibition (Fig. 3e). In addition, mutations at Met183^5.43^ and Asn104^3.36^ also resulted in inactive or loss of function phenotypes. In GPR84, the Asn104^3.36^–Tyr332^6.48^ functions as a non-canonical dual toggle switch^48^, substituting for the classical Trp^6.48^ transmission switch found in class A GPCRs^49^. In the inactive-state structure, Asn104^3.36^ instead forms a hydrogen bond with the ester linkage of the ligand, whereas Tyr332^6.48^ remains in an upward conformation (Fig. 3d, f). As a result, the toggle switch hydrogen bond is disengaged, reflecting a conformationally stabilized inactive state. Notably, additional mutations in residues proximal to the ligand binding site, including Phe170^ECL2^A, Tyr69^2.63^A, and Arg172^ECL2^A, also resulted in loss-of-function or partial loss-of-function phenotypes, whereas Arg172 ^ECL2^K failed to rescue activity (Fig. 3e, f, Supplementary Fig. 10a, and Supplementary Table 2).

### Arginine guidance and hydrophobic gate wall at the ligand entry site

**Fig. 4:**
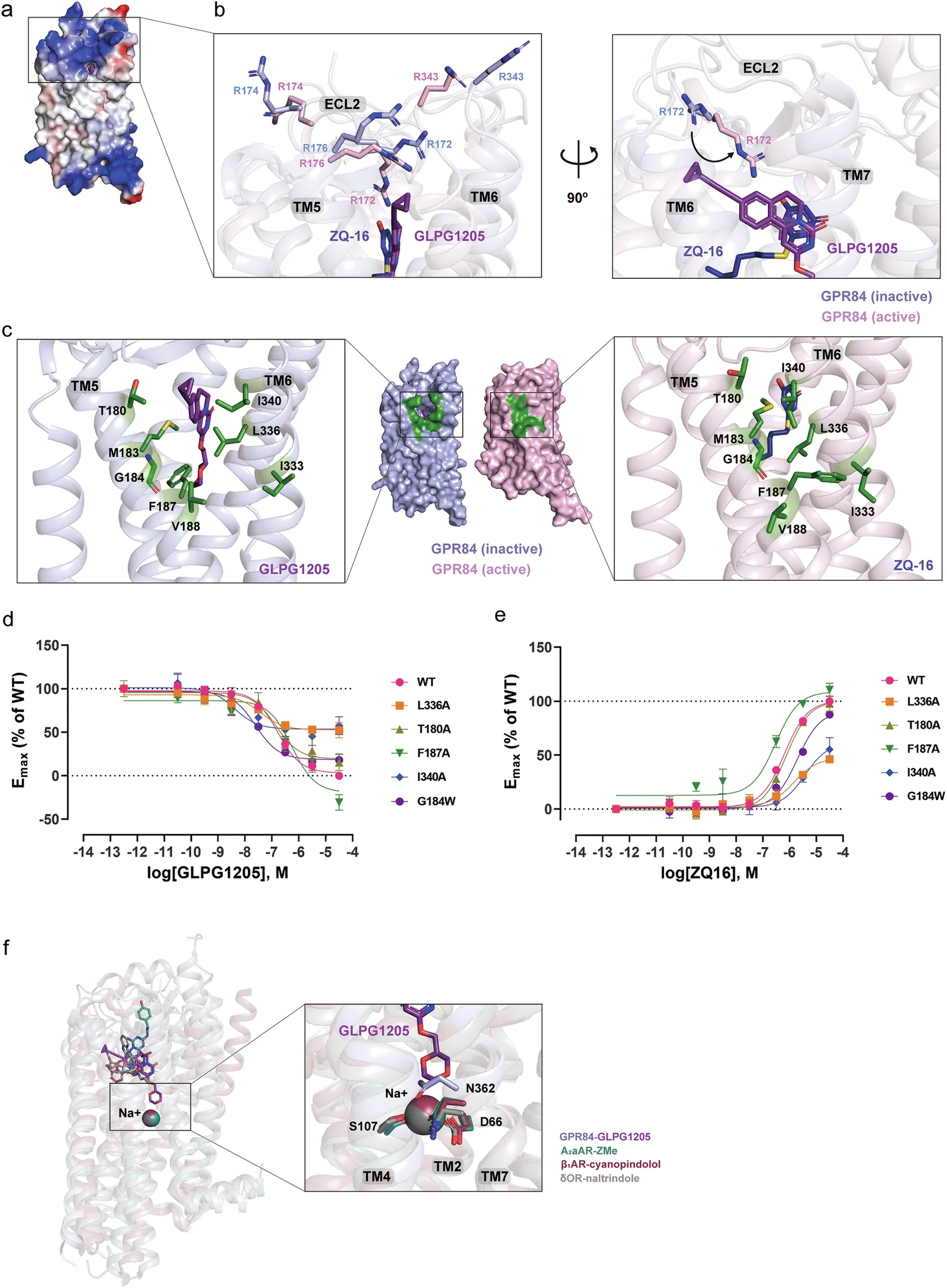
Comparative analysis of inactive and active GPR84. **a** Electrostatic surface potential of inactive GPR84. **b** Differences in the ECL2 conformation between the inactive GLPG1205 (purple)–GPR84 (light blue) and active ZQ-16 (dark blue)–GPR84 (light pink) structures. The arginine cluster is highlighted (left) and the inward movement of R172 is indicated (right). **c** Hydrophobic cavity formation in the GLPG1205-bound structure, involving surrounding residues and surface representation (green, left), compared with the corresponding cavity in the ZQ-16-bound structure (right). **d**, **e** Mutational analysis of hydrophobic residues showing impaired GLPG1205 (left) and ZQ-16 (right) activity in bioluminescence resonance energy transfer assays, which in turn shows preserved activity relative to WT. Data are mean ± sem of *n*  =  3 biological replicates. Dose–response curves were fitted using nonlinear regression. Statistical significance was assessed using one-way analysis of variance. **f** Structural comparison of the GLPG1205-bound receptor with sodium-bound class A GPCRs (A2aAR, PDB: 4EIY, deep teal; β1AR, PDB: 4BVN, raspberry; δOR, PDB: 4N6H, gray). ECL, extracellular loop; *E*_max_, maximum effect; TM, transmembrane; WT, wild-type.

At the distal end, the cyclopropylethynyl tail of GLPG1205 extends into a lateral pocket formed by TM5, TM6, and ECL2 (Fig. 3a). Notably, Arg172^ECL2^, which adopts an inward facing conformation in the active state, undergoes a distinct rotameric shift to interact with the cyclopropyl moiety and engages in van der Waals contacts (Figs. 2b, 3c, and 4b and Supplementary Fig. 12a,b). The extracellular region of GPR84 displays a highly positive charged surface, with arginine residues including Arg172^ECL2^ (Fig. 4a, b). This electropositive surface may function as a “ligand fishing” that captures negatively charged ligands. Given that GPR84 functions as an MCFA receptor, free fatty acids carry a negative charge owing to their carboxylate head groups. These arginine residues are likely to facilitate electrostatic attraction and promote ligand pre-binding orientation near the pocket (Fig. 4a and Supplementary Fig. 5a). These interactions may enable lipid-sensing GPCRs such as GPR84 to guide ligand entry and to position within the membrane region, particularly under conditions of low MCFA abundance^11^.

To experimentally evaluate this proposed arginine-cluster-mediated ligand-fishing mechanism, we performed alanine substitutions of positively charged residues in the extracellular region, followed by BRET assays using ZQ-16, 6OAU, and GLPG1205 (Figs. 2e and 3f, Supplementary Figs. 5b–e and 10a, and Supplementary Table 2). Alanine mutations at Arg20^1.32^, Arg87^ECL1^, Arg94^3.26^, Arg172^ECL2^, Arg176^5.36^, and Arg349^ECL3^ resulted in a pronounced loss-of-function or inactive phenotype across all three ligands, whereas Arg174 ^ECL2^ and Arg344^ECL3^ showed minimal effects. Notably, the loss of signaling was consistently observed across chemically distinct ligands, suggesting that these extracellular arginine residues contribute to a shared mechanism of ligand recognition or initial capture. Collectively, these results support a model in which the arginine-enriched extracellular surface functions as an electrostatic guidance platform that facilitates ligand recruitment before engagement with the orthosteric pocket.

The lipophilic tail also engages hydrophobic interactions with Met183^5.43^, Gly184^5.44^, Phe187^5.47^, Leu336^6.52^, and Ile340^6.56^ (Fig. 4c). Although Ala343^6.59^, Val188^5.48^, and Ile333^6.49^ do not directly interact with antagonist, they constitute a lipophilic wall. Lateral ligand entry through TM interfaces has been reported in various lipid-sensing GPCRs^50–54^. Among class A GPCRs, the most frequently observed lateral ligand entry sites are located between TM1 and TM7 exemplified by cannabinoid receptor 1 (CB1) and sphingosine-1-phosphate receptor 1 (S1PR1) and between TM3 and TM4, as seen in FFAR1 and corticotropin-releasing factor receptor 1 (CRF₁R). In GPR84, our structure reveals a distinct lateral opening between TM5 and TM6 that is absent in the active-state conformation, suggesting a potential ligand entry pathway (Fig. 4c). Alanine substitutions at these hydrophobic gate residues markedly impaired the GPR84 signaling in the BRET assay (Fig. 4d, e, Supplementary Fig. 10a, and Supplementary Table 2). For GLPG1205, mutations at Leu336^6.52^A, Thr180^5.40^A, Phe187^5.47^A, Ile340^6.56^A, and Gly184^5.44^W resulted in loss-of-function phenotypes. By contrast, the effects of these mutations were less pronounced for ZQ-16, with Leu336^6.52^A and Ile340^6.56^A showing partial loss of function, Thr180A displaying WT-like activity, and Gly184^5.44^W exhibiting reduced potency. Notably, Phe187^5.47^A resulted in increased potency.

Notably, Phe335^6.51^ induces a steric clash with both the core and the tail of GLPG1205, thereby preventing the TM5–TM6 region from adopting the more open conformation compared with the AlphaFold-predicted model (Supplementary Fig. 3c,d). Consequently, the lateral entry site is more clearly defined in our experimental structure. This TM5–TM6 vestibule, supported by previous MD simulations, may serve as a selective gateway for lipophilic ligand^50^. Our MD simulations revealed that GLPG1205 migrates from the lateral gate into the orthosteric binding site, indicating that this lateral gate serves as the ligand entry pathway (Supplementary Fig. 6c).

The TM5–TM6 interface hydrophobic residues Met183^5.43^, Val188^5.48^, and Leu191^5.51^ on TM5 and Ala329^6.45^, Ile333^6.49^, Leu336^6.52^, Ile340^6.56^, and Ala343^6.59^ on TM6 form a hydrophobic “gateway” that facilitates lateral ligand access (Fig. 4c). Alanine substitutions significantly impaired GPR84 activation and inhibition in the BRET assay (Fig. 4d, e, Supplementary Fig. 10a, and Supplementary Table 2). Previously reported MD simulations of lipid entry pathways in rhodopsin GPCR are regulated by phenylalanine residues that act as gatekeepers; Phe208^5.43^, Phe273^6.56^, and Phe276^6.59^ obstruct lipid insertion by forming a tightly packed aromatic barrier^50^. In GPR84, analogous positions are occupied by Met183^5.43^, Ile340^6.56^, and Ala343^6.59^, suggesting a gating mechanism that relies on hydrophobic interactions. To further assess whether this hydrophobic gateway is conserved across different agonists, we performed additional mutagenesis analysis using 6OAU (Supplementary Fig. 5e). Mutations at Leu336^6.52^A and Thr180^5.40^A exhibited effects comparable to those observed with ZQ-16, whereas Gly184^5.44^W and Ile340^6.56^A showed a partial loss-of-function phenotype in response to 6OAU relative to ZQ-16. These results suggest that although the overall architecture of the ligand entry pathway is conserved, certain residues contribute in a ligand-dependent manner, supporting a shared but adaptable mechanism of ligand access.

### Capping the sodium-binding pocket by GLPG1205

**Fig. 5:**
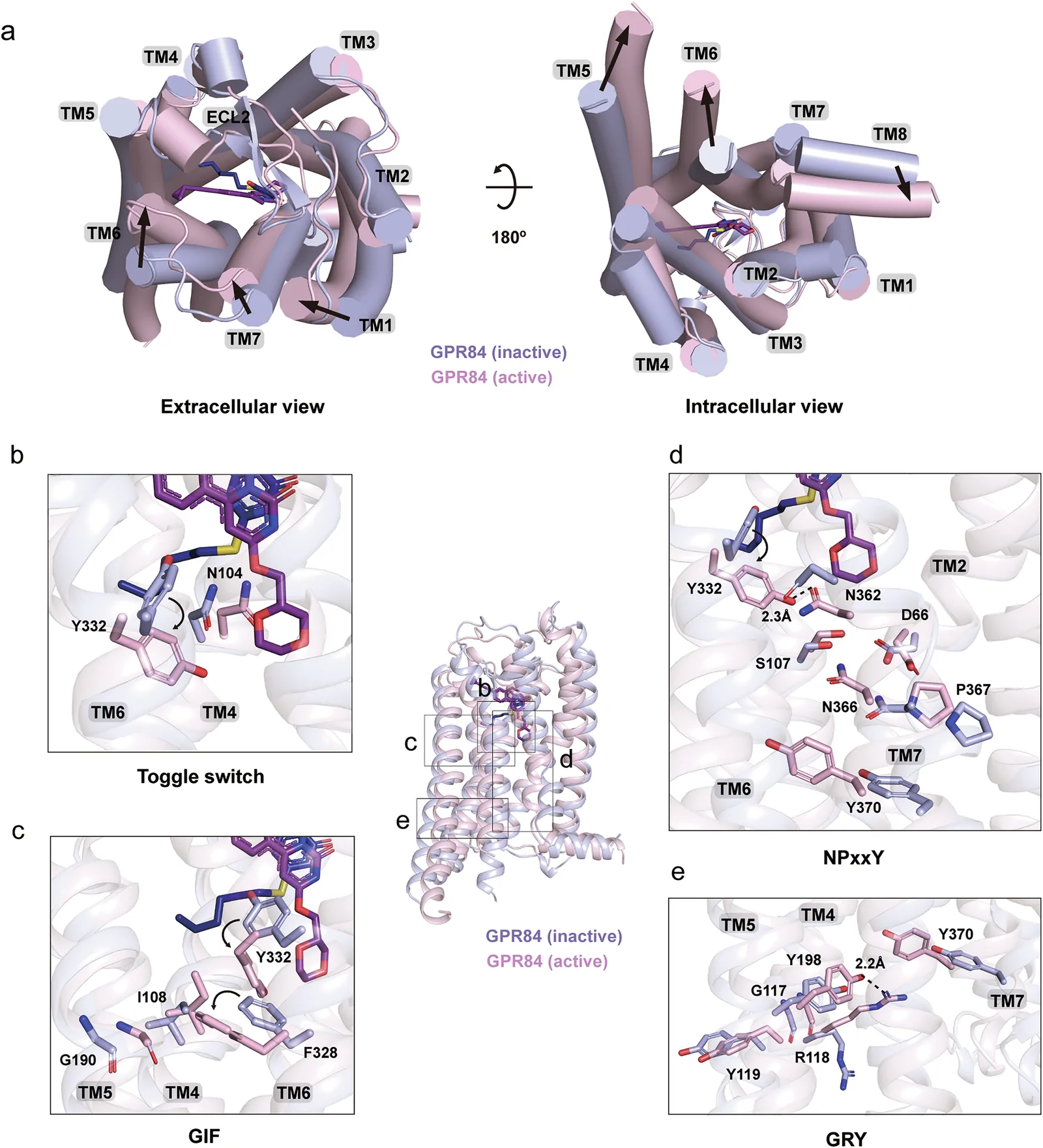
Conformational rearrangements and conserved motifs in inactive and active GPR84. **a** Extracellular and intracellular views of superimposed inactive GLPG1205 (purple)-GPR84 (light blue) and active ZQ-16 (dark blue)-GPR84 (light pink) structures, highlighting major helical movements. Rearrangement of the toggle switch (part **b**). **c** GIF motif. **d** NPxxY motif. **e** Non-canonical GRY motif (hydrogen bonds indicated by black dashed lines). ECL, extracellular loop; TM, transmembrane.

The dioxane head group of GLPG1205 is deeply inserted into the TM cavity, adjacent to TM2 and TM3 (Fig. 3a, d). Notably, Tyr69^2.53^ forms a T-shaped CH–π interaction with the dioxane ring, as supported by strong and continuous electron density. This interaction positions the dioxane moiety near the canonical sodium-binding pocket, typically formed by a conserved Asp^2.50^ and Asn^7.45^, Asn^7.49^, and Ser/Thr^3.39^, which coordinates sodium ion via hydrogen bond network^55,56^. The van der Waals surfaces of adjacent residues Asn104^3.36^, Ser103^3.35^, Asp66^2.50^, Ser107^3.39^, Asn362^7.45^, Phe328^6.44^, and Gly363^7.46^ form a hydrophilic interface around the ligand head (Fig. 5b, d).

Structural alignment with sodium-bound GPCR structures, such as A2aAR (PDB: 4EIY), beta1 adrenoceptor (β₁AR, PDB: 4BVN), and δ-opioid receptor (δOR, PDB: 4N6H), reveals that the network maintains conserved inter-residue distances that support sodium ion coordination (Fig. 4f and Supplementary Fig. 7a). By contrast, the antagonist-bound GPR84 structure exhibits a disrupted sodium binding geometry. Although the equivalent residues Asp66^2.50^, Asn362^7.45^, and Ser107^3.39^ are conserved, Asn362^7.45^ is markedly displaced upward, resulting in significantly extended distances to Asp66^2.50^ and Ser107^3.39^, incompatible with stable ion binding. This aberrant configuration probably arises from steric hindrance by the dioxane head of GLPG1205, which caps the sodium conduit and prevents coordination at Asn362^7.45^. Although Asn366^7.49^ maintains canonical spacing for sodium binding site, solvent-accessible surface analysis further supports the notion that the dioxane ring occludes sodium entry (Supplementary Fig. 7b). This mechanism likely contributes to its pharmacological antagonism by collapsing the allosteric ion site essential for receptor activation in class A GPCRs^55–57^ (Supplementary Fig. 7c). The resulting interaction footprint defines key pharmacophoric features required for antagonistic activity.

### Structural differences between inactive and active states

To elucidate the activation mechanism of GPR84, we performed a comparative analysis of the inactive and active states. Superimposition of the TM helices yields a Cα RMSD of 2.25 Å, with conformational changes observed in TM5, TM6, and TM7, hallmarks of class A GPCR activation^49,58^ (Fig. 5a and Supplementary Fig. 8a).

In the active-state structure, ZQ-16 adopts the lowest binding pose, driving Tyr332^6.48^ into a lower position and establishing the closest Tyr332^6.48^– Asn362^7.45^ interaction (Figs. 2d and 5b). At the same time, thioether linker forms a polar interaction with Asn104^3.36^, disrupting the Asn104^3.36^–Tyr332^6.48^ hydrogen bond (Fig. 5b). By contrast, 6OAU maintains moderate interactions with Asn104^3.36^ and Tyr332^6.48^, whereas LY237 fails to contact Asn104^3.36^ (refs. ^8,48^). This displacement induces a downward swing of Tyr332^6.48^ in the ZQ-16-bound structure, which sterically displaces Phe328^6.44^ outward and reorients Ile108^3.40^ of the conserved PIF motif^49,58^ (Fig. 5c, d and Supplementary Fig. 9b). These conformational rearrangements are accompanied by an inward movement of TM5 and outward displacement of TM6, forming a cytoplasmic cavity compatible with G protein engagement (Fig. 5a and Supplementary Fig. 8a). Simultaneously, Tyr332^6.48^ establishes a new hydrogen bond with Asn362^7.45^, promoting the inward displacement of TM7 (Fig. 5d). This reorganization is further supported by reduced distances between Asn362^7.45^ and Ser107^3.39^, as well as between Asn366^7.49^ and Ser107^3.39^ and Asn362^7.45^. Meanwhile, Asn366^7.49^ shifts away from Asp66^2.50^, disrupting the canonical hydrogen bond and contributing to a collapse of the sodium-binding pocket^55–57^. Unlike typical class A GPCRs, in which Asn^7.45^ commonly forms a hydrogen bond with Asp^2.50^, no such interaction is observed in either of the GPR84 states. Subsequently, Asn366^7.49^ forms a new hydrogen bond with Tyr370^7.53^, completing the NPxxY motif and facilitating inward movement of TM7^49^. This rearrangement is accompanied by inward shifts of Pro367^7.50^ and Tyr370^7.53^. The repositioned Tyr370^7.53^ also enables new polar contacts with Arg118^3.50^ from the GRY motif (Gly117^3.49^, Arg118^3.50^, and Tyr119^3.51^), a non-canonical variant of the class A DRY motif (Fig. 5e).

TM1–TM3 shift inward at the extracellular side and TM8 are repositioned closer to TM1, whereas TM4 remains largely unchanged (Fig. 5a and Supplementary Fig. 8a). ICL2 becomes more upright from a relaxed helical form to engage Gαi, and ICL1 transitions from a flexible loop upon activation. Overall, the distinct reconfiguration observed in the structure provides a mechanistic framework for understanding GPR84 activation at the atomic level.

### Comparison of diverse agonists modulate GPR84 activation

Consistent with the canonical class A GPCRs, the α5 helix of Gαi inserts into the cytoplasmic cavity of GPR84 and interacts with TM2, TM3, TM5, and TM6, and is displaced upward by 1.3 Å toward the receptor core, forming an extensive hydrophobic interface with the G protein^49^ (Supplementary Fig. 9a).

Specifically, Gαi residues Ile344, Leu348, Cys351, Leu353, and Phe354 engage hydrophobic side chains on TM3, TM5, and TM6 of GPR84 (Supplementary Fig. 9c). In contrast to previous GPR84–Gαi complexes, in which Phe354 of Gαi participates in aromatic stacking with Phe314^6.30^, our structure reveals that Phe314^6.30^ is rotated outward and does not engage in this interaction^48^ (Supplementary Fig. 9d,c). Additionally, Cys351 α5 helix of Gαi forms a hydrogen bond with Arg118^3.50^ of DRY motif (Supplementary Fig. 9e). The elongated TM5 allows Tyr215^5.75^ to engage in π–π interactions with Phe334 and His322 of Gαi and hydrogen bond Gln333 of Gαi (Supplementary Fig. 9f). Notably, a unique salt bridge between Lys345 of Gαi and Glu313^6.29^ is observed (Supplementary Fig. 9h). Upon ZQ-16 binding, TM8 is displaced further toward TM1, leading to a hydrogen bond between Gln376^8.49^ and Asp350 of Gαi (Supplementary Fig. 9g). This also disrupts conserved Gβ–receptor interactions. Specifically, the salt bridge between Asp312 of Gβ and Lys50ICL1/Arg387^8.60^ of GPR84, as well as the cation–π interaction between Phe292 of Gβ and Lys386^8.59^, are no longer observed.

The αN terminus of Gαi positioned closer to the receptor compared with other GPR84-active structure (Supplementary Fig. 9a). Unlike most class A GPCRs, which typically feature a hydrophobic residue at position 34.51, GPR84 harbors an Lys126^ICL2^ at this site, mediating interactions with the αN helix of Gαi^8,48^ (Supplementary Fig. 9i). However, in the ZQ-16-bound structure, no direct contact is observed between Lys126^ICL2^ and Asp93 of Gαi. Phe128^ICL2^ and Phe132^ICL2^ engage in tight packing interactions with TM2–TM4, including Phe55^2.39^, Leu113^3.45^, Leu116^3.48^, Leu120^3.52^, Leu121^3.53^, and Ile137^4.42^ (Supplementary Fig. 9j). This network is maintained across all GPR84 agonist complexes and likely supports the upward displacement of ICL2 (Supplementary Fig. 8a). These comparative analyses underscore that individual agonists may differentially stabilize active conformations of GPR84.

### Macrophage polarization and cytokine analysis

**Fig. 6:**
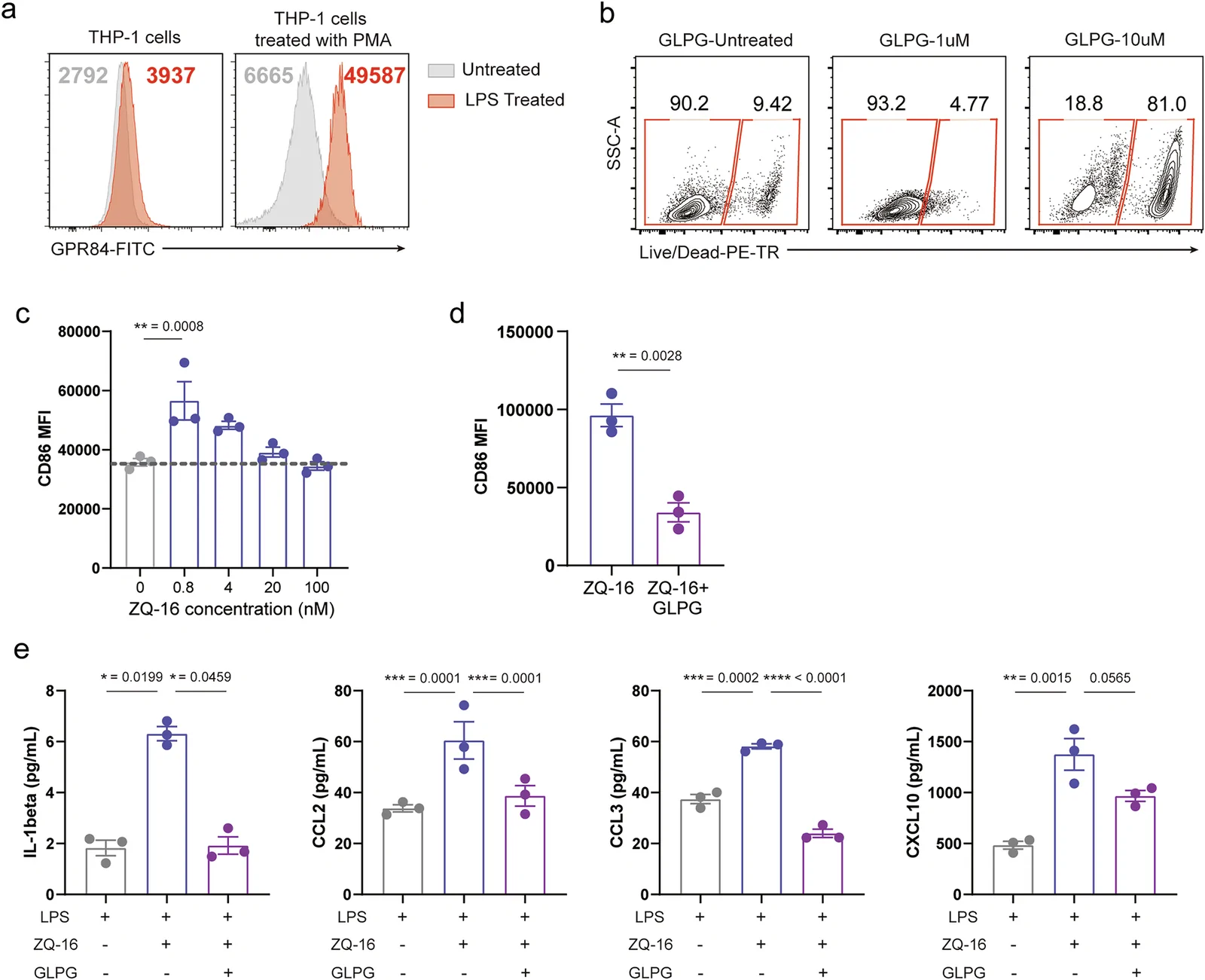
Functional effects of GPR84 modulation in THP-1 cells assessed by flow cytometry and cytokine analysis. **a** GPR84 expression from THP-1 and phorbol 12-myristate 13-acetate (PMA)-induced THP-1 cells before and after lipopolysaccharide (LPS) (100 ng/ml) treatment. Numbers indicate mean fluorescence intensity (MFI). **b** FACS plots showing percentages of live (left) and dead (right) cells in response to different doses of GLPG1205 after treating THP-1 cells for 16 h. **c** Bar graph showing CD86 MFI at different ZQ-16 concentrations. **d** Bar graph showing CD86 MFI LPS-stimulated, PMA-induced THP-1 cells treated with ZQ-16 (0.8 nM) alone or in combination with GLPG1205 (1 μM). **e** Cytokine concentrations in culture supernatants under indicated treatment conditions. The concentrations for ZQ-16 and GLPG1205 are 0.8 nM and 1 μM, respectively. Statistical significance for parts **c**–**e** was determined by one-way analysis of variance with Tukey’s multiple comparisons test. Except for FACS data in panel **b** which was conducted once, all other data are representative of two independent experiments.

LPS induces transcription of multiple pro-inflammatory genes, including cytokines and receptors involved in innate immune responses. Consistent with this, previous studies have shown that GPR84 expression is upregulated in monocytes and macrophages upon LPS stimulation^59^. The expression of GPR84 after LPS stimulation was assessed in human monocytic cell line THP-1. Although mean fluorescence intensity value showed some increment, the GPR84 expression was not substantial after 24 h stimulation of LPS. Therefore, we differentiated THP-1 into macrophages using PMA and subsequently stimulated them with LPS. The analysis demonstrated that GPR84 expressions were more pronounced than in monocytes and clearly detectable after LPS stimulation (Fig. 6a). As GPR84 expression was induced by LPS, we next examined the appropriate dosage of GLPG1205, as previous studies have reported variable concentrations for its effect^8,60^. When THP-1 cells were treated with GLPG1205 in combination with ZQ-16 for 16 h, 10 μM of GLPG1205 resulted in significant impact on cell viability, whereas 1 μM maintained cell viability (Fig. 6b). Therefore, we set the GLPG1205 concentration at 1 μM for subsequent experiments using PMA-induced THP-1 to allow assessments of differential surface molecule expression in response to GPR84 agonist–antagonist interactions.

Given that 6OAU showed a plateau in the pro-phagocytic ability of BMDMs at higher concentrations^8^ and that ZQ-16 binds more strongly to GPR84 than 6OAU, we hypothesized that a lower concentration of ZQ-16 would be sufficient to induce activation in PMA-treated THP-1 cells. Analysis of the mean fluorescence intensity showed that 0.8 nM ZQ-16 was sufficient to modulate CD86 expression, with no further increase observed at higher concentrations. (Fig. 6c). The presence of GLPG1205 effectively abrogated this effect when treated along with ZQ-16 (Fig. 6d).

In addition to the expression of activation markers, ZQ-16 enhanced the functional capacity of macrophages to produce and secrete inflammatory cytokines. In culture supernatants, ZQ-16 stimulation increased the levels of IL-1β, CCL2, CCL3, and CXCL10 when GPR84 expression had been upregulated by LPS. However, similar to its effect on CD86 expression, GLPG1205 markedly reduced the ZQ-16-induced production of these cytokines (Fig. 6e).

## Discussion

In this study, we determined cryo-EM structures of GPR84 in its inactive and active states, complemented by functional assays that collectively establish the molecular basis of receptor modulation. The inactive-state structure reveals how GLPG1205 stabilizes GPR84: its dioxane head group directly caps the canonical sodium-binding site formed by Asp^2.50^, Ser^3.39^, and Asn^7.45^. In most class A GPCRs, sodium binding stabilizes the inactive state and modulates receptor activation by bridging TM helixes^55,56^. Structures of A₂AAR, β₁AR, and δOR have established sodium coordination as a conserved feature among class A GPCRs ^55–57^. By displacing Asn^7.45^ and disrupting the hydrogen bond network, GLPG1205 abolishes sodium coordination. This mechanism may provide a framework for the rational design of GPR84 antagonists that target allosteric sites. By contrast, the ZQ-16-bound structure highlights a noncanonical activation pathway involving a distinctive toggle switch. Most class A GPCRs use Trp^6.48^ as the primary transmission switch^49,58^; however, in GPR84, this role is fulfilled by a dual toggle comprising Asn104^3.36^ and Tyr332^6.48^. ZQ-16 disrupts the Asn104^3.36^–Tyr332^6.48^ hydrogen bond and repositions Tyr332^6.48^ downward, initiating outward displacement of TM6 and inward movement of TM7. This cascade propagates to the PIF, NPxxY, and GRY motif, reorganizing the cytoplasmic cavity for G protein engagement. A notable feature observed across our structures is the conformational plasticity of ECL2. In the GLPG1205-bound structure, ECL2 adopts a rigid, β-strand-stabilized lid, consistent with previously reported GPR84 structures^50–52^. Conversely, in the ZQ-16 complex, ECL2 becomes disordered. This structural plasticity is consistent with earlier MD simulations proposing that ECL2 facilitates ligand entry^8,48^. Our findings emphasize that ECL2 conformational states are not static but dynamically coupled to ligand entry and receptor activation. A similar electrostatic guidance mechanism has been observed in chemokine receptors such as CXCR4, in which the negatively charged ligand CXCL12 is initially captured by positively charged extracellular regions before engaging the orthosteric pocket, consistent with a two-step binding mechanism^61^.

Our inactive state structure also reveals a lateral opening between TM5 and TM6 that becomes occluded in the active conformation. TM5–TM6 hydrophobic residues such as Met183^5.43^, Leu336^6.52^, and Ile340^6.56^, which collectively form a “hydrophobic gateway” and functional mutagenesis, demonstrated that disrupting these residues abolished antagonist activity, validating the functional relevance of this portal. In addition, membrane-embedded ligand entry through a lateral opening between TM5 and TM6 has been proposed in rhodopsin, in which MD simulations revealed that phenylalanine residues act as gatekeepers by forming a tightly packed aromatic barrier^50^. Interestingly, the GLPG1205-bound inactive structure revealed extra density in the region between TM5 and TM6 (Supplementary Fig. 11a). MD simulations indicated potential lipid interactions at this site, suggesting that it could accommodate a hydrophobic moiety POPC (Supplementary Fig. 6b,c). To further explore this possibility, we tested candidate molecules by fitting them into the electron density map. Given the lipid composition of sf9 insect cells, long chain fatty acids, and cholesterol hemisuccinate (CHS)^50^, which were added during purification, these components were plausible candidates (Supplementary Fig. 11b,c). However, in the human physiological context, it is conceivable that MCFAs, the endogenous ligands of GPR84, may enter the receptor via this lateral TM5–TM6 pathway (Supplementary Fig. 11c).

Comparisons across agonists reveal that differences in ligand tail length and head group chemistry translate into distinct stabilization of active conformations. ZQ-16 engages deeply with toggle switch residues, correlating with its enhanced ability to promote cytokine production in macrophages. Functional validation in THP-1 macrophages demonstrated that ZQ-16 robustly induces CD86 expression and cytokine secretion, whereas GLPG1205 abrogates these effects. These results link structural observations to immune phenotypes and provide mechanistic support for pharmacological manipulation of GPR84 in inflammatory settings. Consistent with this interpretation, the structural features identified in this study — including the conformational plasticity of ECL2, the electropositive extracellular surface, and the lateral opening between TM5 and TM6 — suggest mechanisms for ligand capture and membrane-embedded entry under altered lipid conditions. Dysregulated GPR84 activation has been implicated in diseases such as IPF and inflammatory bowel disease, both of which involve macrophage-mediated inflammation. Because GPR84 appears to be regulated primarily through receptor expression and ligand-dependent activation rather than pathogenic mutations, these structural insights provide a framework for the rational design of antagonists aimed at suppressing excessive receptor activation. GPR84 has been implicated in both protective and pathogenic roles across neurodegeneration, fibrosis, and metabolic disorders^16–24^. Our data explain how antagonists such as GLPG1205 suppress pro-inflammatory macrophage responses, consistent with its antifibrotic activity in preclinical models^26–30^. Conversely, agonists may amplify immune responses, potentially offering therapeutic opportunities in contexts in which macrophage activation is beneficial, such as infection or neurodegenerative disease^16–19^. These dual roles highlight the importance of pathway selective modulators tailored to disease context.

Many orphan GPCRs exhibit low affinity ligand interactions and lack clear endogenous agonists, complicating pharmacological targeting^48^. By characterizing the structural determinants of both inactivation and activation, our study establishes design principles for selective modulation. In addition, MD simulations revealed a movement of GLPG1205 within the binding pocket (Supplementary Fig. 6a–c), providing a potential explanation for its limited efficacy in IPF trials and underscoring the need to develop improved antagonist scaffolds. Notably, the sodium-binding pocket represents a potential pharmacophore that can be occluded by synthetic antagonists, offering an underexplored avenue for selective drug development. Together, these findings provide structural insights that may enable the rational design of pathway-selective GPR84 modulators. Although comprehensive, this study has several limitations, including the absence of structures bound to endogenous ligands and reliance on synthetic agonists and antagonists. Moreover, the functional assays were performed exclusively in THP-1-derived macrophages, and the diversity of GPR84 signaling across cell types remains to be fully characterized. Future studies should therefore resolve structures of GPR84 bound to endogenous MCFA ligands, investigate ligand-entry pathways in greater detail, and assess signaling diversity — including β-arrestin recruitment — to clarify the therapeutic potential of GPR84 modulation.

## Supplementary information

Supplementary information

Structure file

## Data Availability

Coordinates and structure factors for the inactive and active forms of GPR84 have been deposited in the Protein Data Bank (PDB) under accession codes 9V6T (inactive) and 9UPY (active), respectively. The corresponding cryo-EM maps are available in the Electron Microscopy Data Bank (EMDB) under accession codes EMD-64807 (inactive) and EMD-64407 (active). All other data supporting the findings of this study are available from the corresponding author upon reasonable request. The MD source data are available at Zenodo (https://doi.org/10.5281/zenodo.17128240).
