# Supplementary material for: Pharmacological modulation of GPR84 revealed by dual states structures and immune functional assays
Source: Exp Mol Med. 2026 Sep 10;58(9):3127–40. doi: 10.1038/s12276-026-01841-w (PMC13633242; doi:10.1038/s12276-026-01841-w)
Supplement: Supplementary file 1 [file 12276_2026_1841_MOESM1_ESM.pdf]

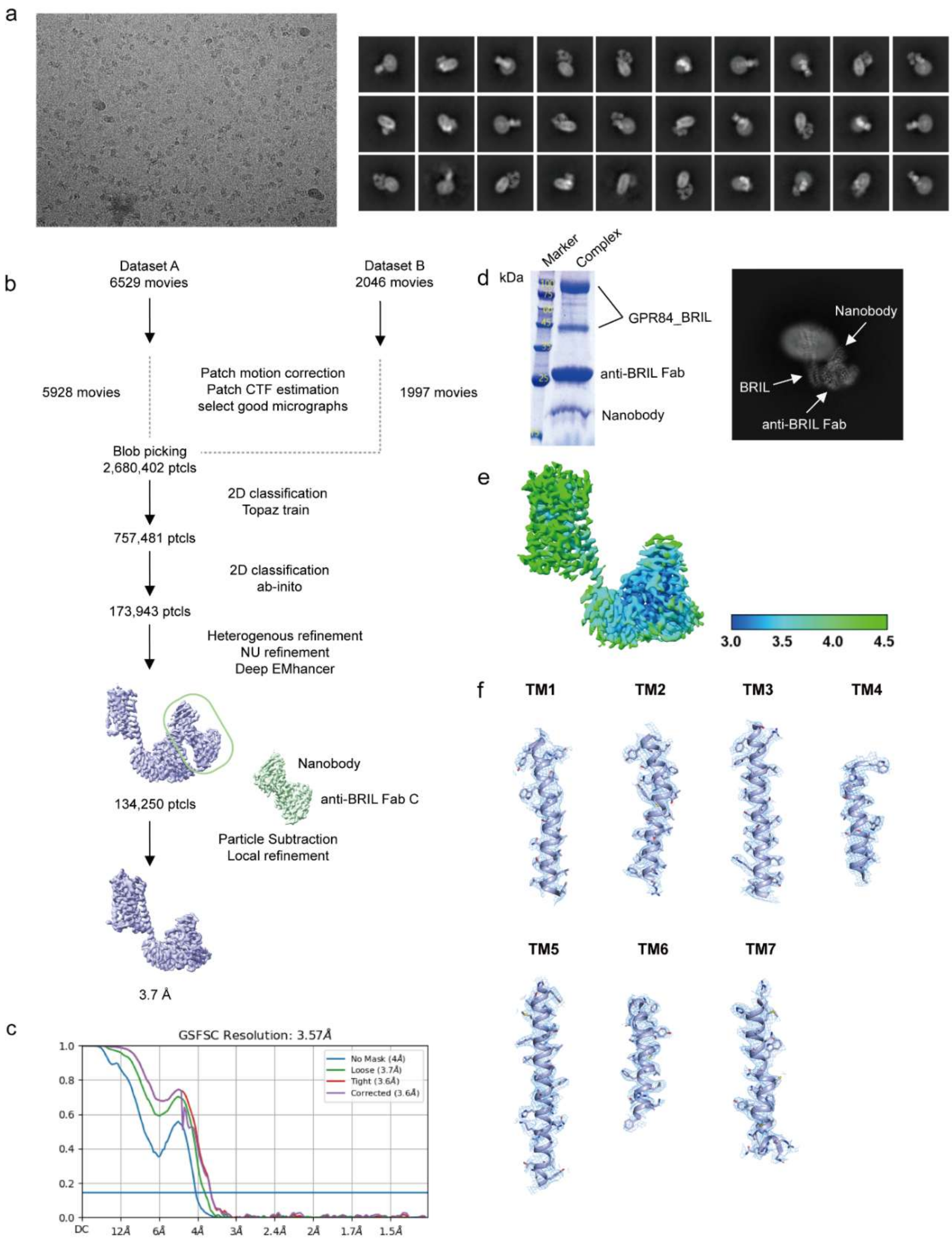

Supplementary Fig. 1 a, Representative cryo-EM micrograph and selected 2D class averages. b, Image processing workflow. From 8,529 movies (Dataset A) and 2,046 movies (Dataset B), 2D classification, ab-initio reconstruction, and heterogeneous refinement yielded a final map at 3.7 Å resolution after particle subtraction of anti-BRIL Fab C and Nanobody and local refinement. c, Gold standard Fourier shell correlation (GSFSC) curve indicating an overall resolution of 3.57 Å at the 0.143 cutoff. d, SDS PAGE gel of the purified complex (left), EM image showing BRIL, anti-BRIL Fab, and nanobody (Right). e, Local resolution map of the final reconstruction, colored from 3.0 Å to 4.5 Å. f, Representative density maps for individual transmembrane helices (TM1-TM7).

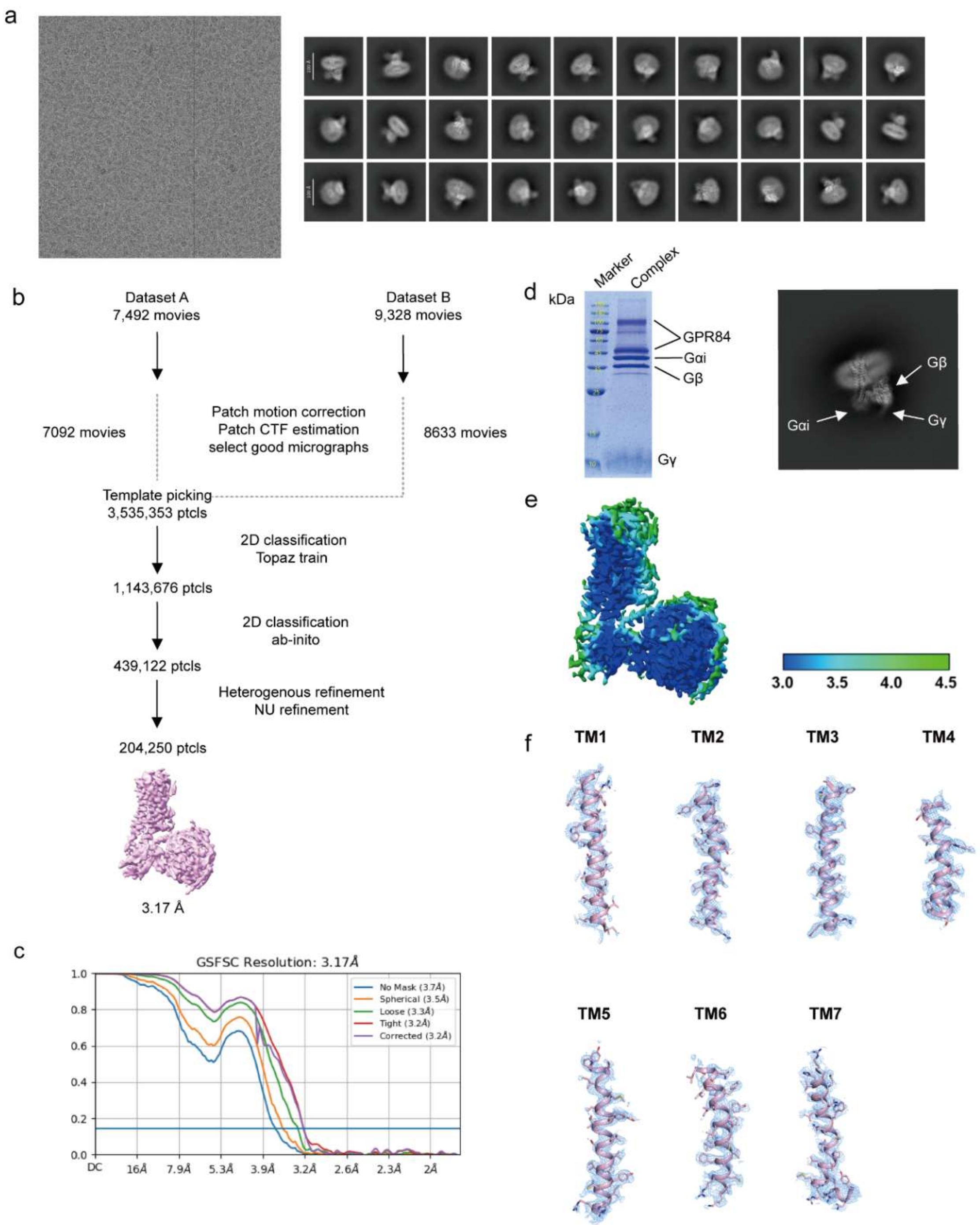

Supplementary Fig. 2 a, Representative cryo-EM micrograph and selected 2D class averages. b, Image processing workflow. From 7,492 movies (Dataset A) and 9,328 movies (Dataset B), 2D classification, ab initio reconstruction, and heterogeneous refinement yielded a final map at 3.17 Å resolution. c, Gold standard Fourier shell correlation (GSFSC) curve indicating an overall resolution of 3.17 Å at the 0.143 cutoff. d, SDS PAGE gel of the purified complex (left), EM image showing Gαiβγ (Right). e, Local resolution map of the final reconstruction, colored from 3.0 Å to 4.5 Å. f, Representative density maps for individual transmembrane helices (TM1-TM7).

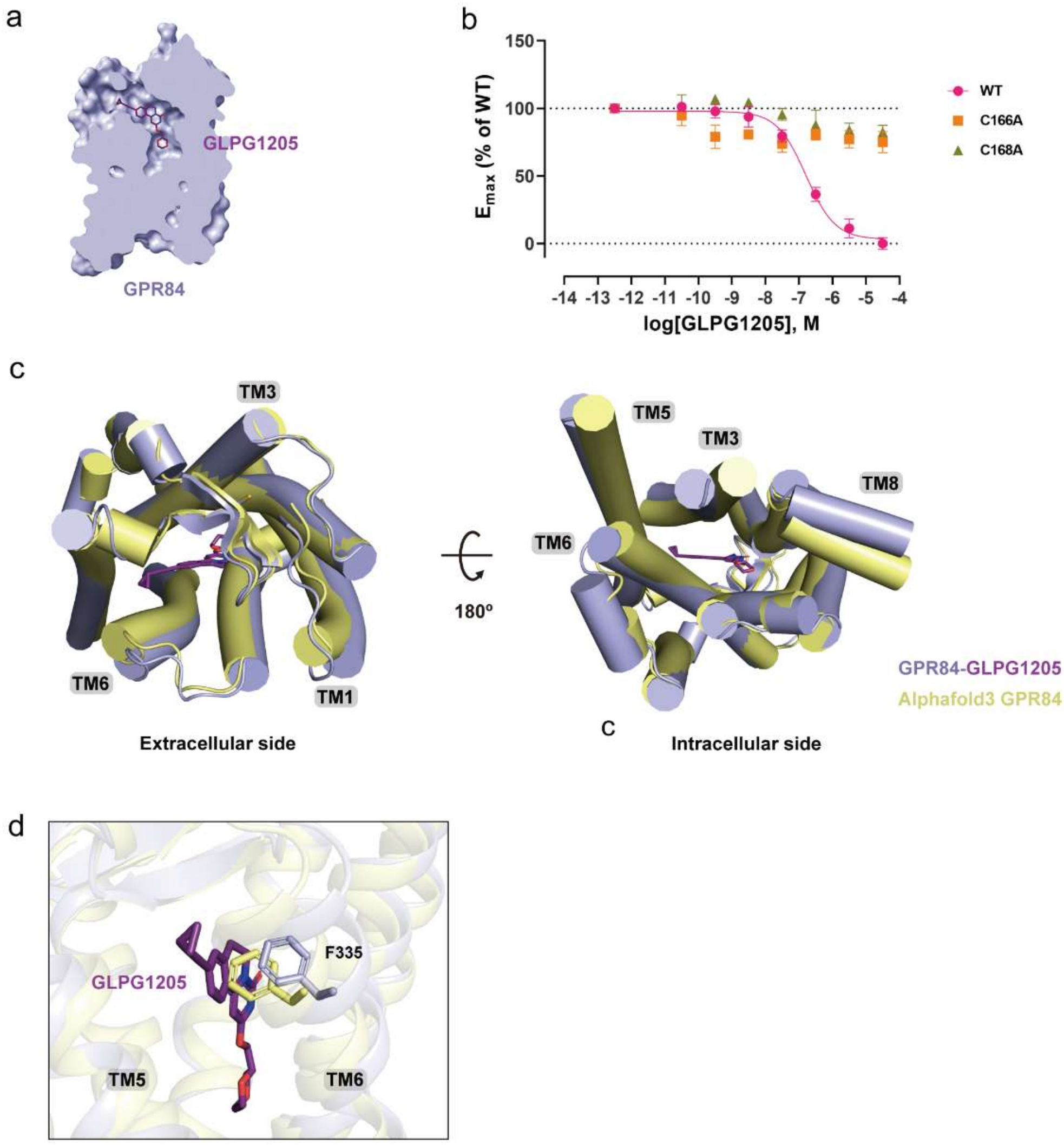

Supplementary Fig. 3 a, Ligand binding pocket of inactive state GPR84(light blue) bound to GLPG1205 (purple). b, BRET assay of alanine mutants (C16A, C168A), showing loss of GLPG1205 responsiveness compared with WT. Data are mean ± s.e.m. of $n = 3$ biological replicates. Dose response curves were fitted using nonlinear regression. Statistical significance was assessed using one-way ANOVA. c, Superimposition of the GLPG1205-GPR84 structure with the AlphaFold3 predicted inactive model (yellow), viewed from the extracellular (left) and intracellular (right) sides. d, Close up view of the TM5-TM6 interface with GLPG1205 with residue F335.

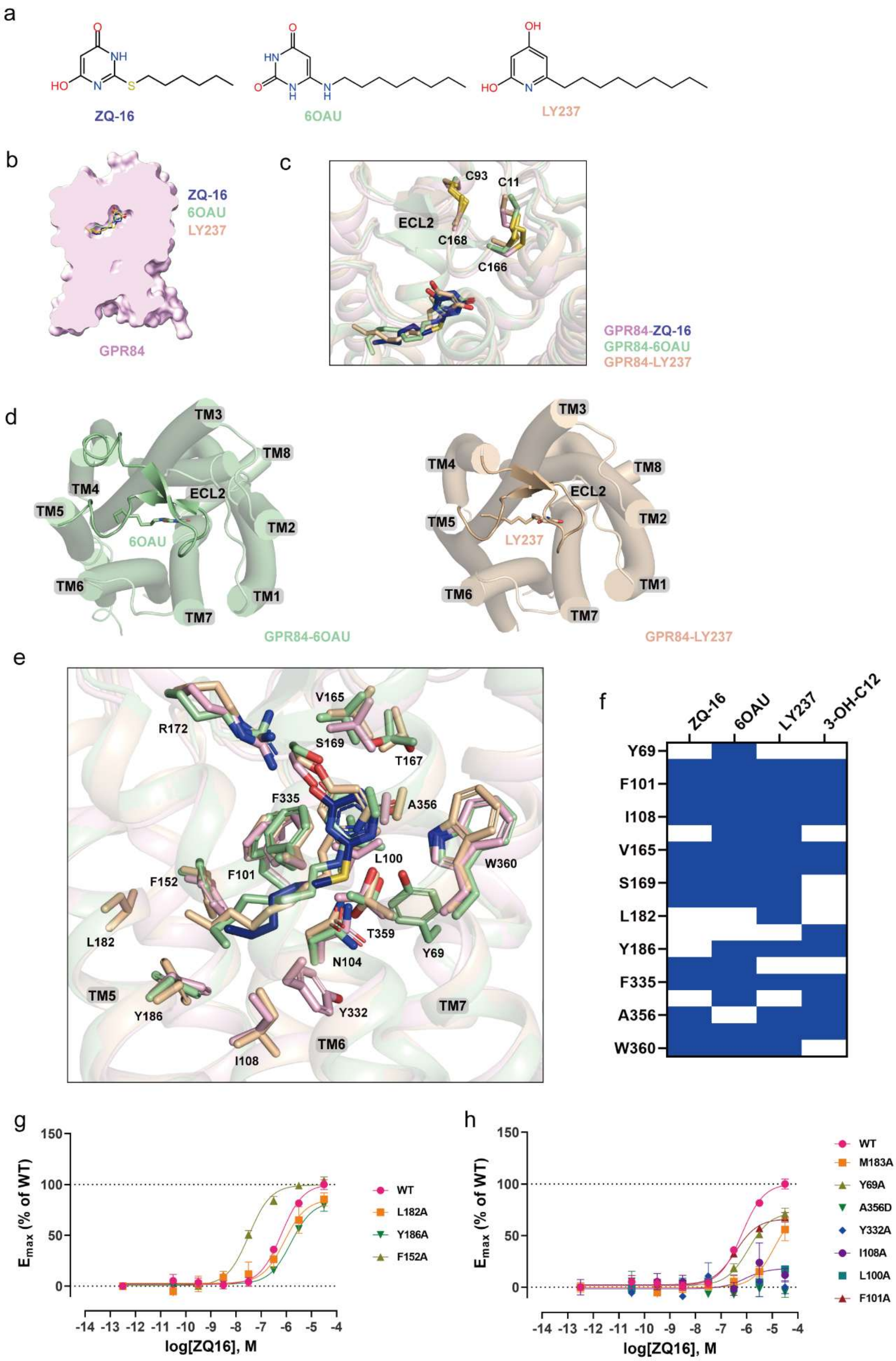

Supplementary Fig. 4 a, Chemical structures of ZQ-16, 6-OAU, and LY237. b, Ligand binding pocket of active state GPR84 with ZQ-16, 6-OAU, (light green, PDB: 8G05) and LY237 (light orange, PDB: 8J19). c, Superimposition of the three agonists bound GPR84 structures, highlighting the disulfide bonds. d, ECL2 conformations of 6-OAU and LY237 bound GPR84 (extracellular view). e, Overlay of ZQ-16, 6-OAU, and LY237 binding modes, illustrating conserved polar

and hydrophobic interactions with residues including. f, Barcode representation of ligand receptor interactions for GPR84 agonists. Residues within 4 Å of each ligand (LY237, 3-OH-C12 FFA, 6-OAU, and ZQ-16) are shown in blue. g,h, BRET assays of key hydrophobic wall mutants, showing impaired ZQ-16 induced activation compared with WT. Data are mean ± s.e.m. of $n = 3$ biological replicates. Dose response curves were fitted using nonlinear regression. Statistical significance was assessed using one-way ANOVA.

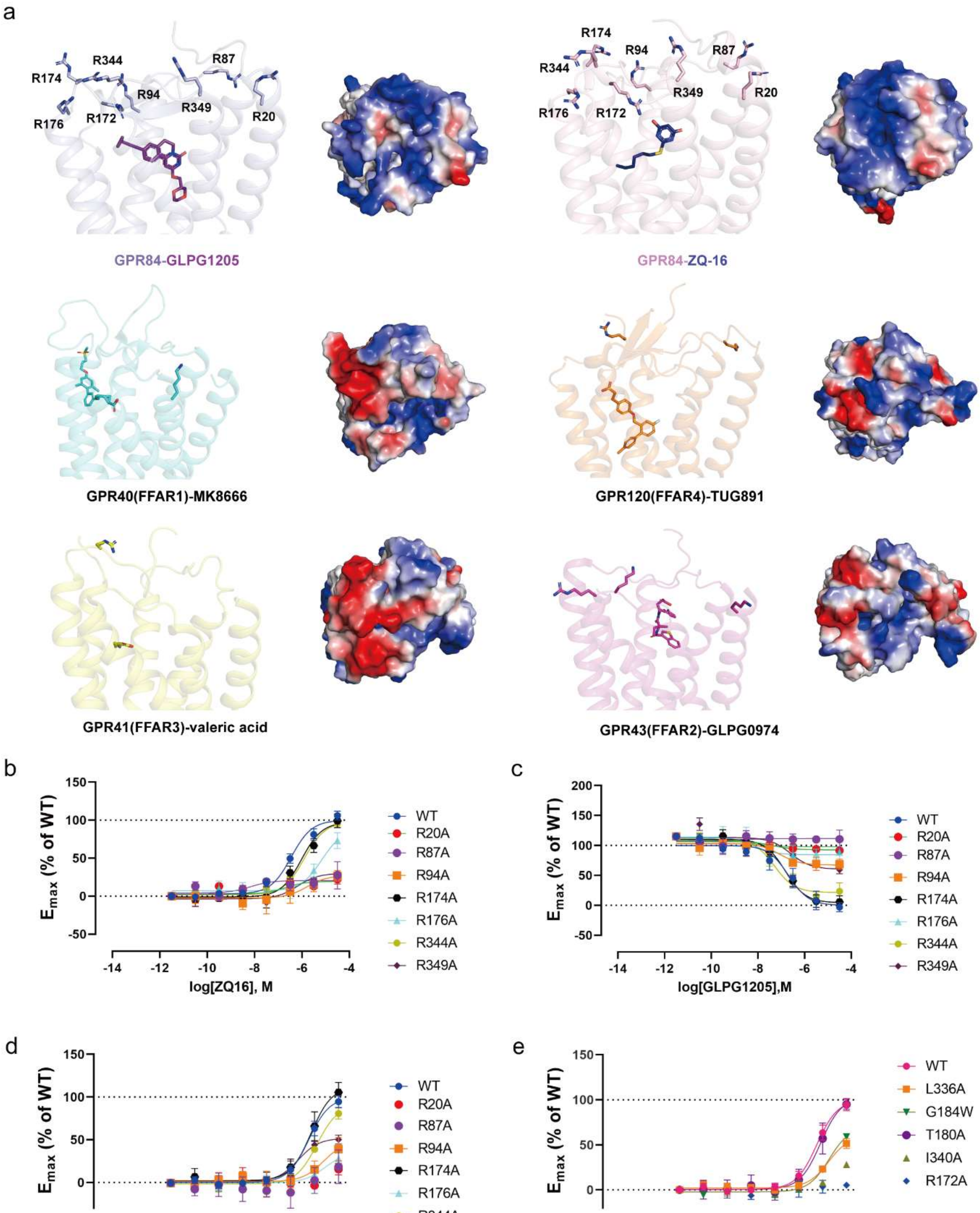

Supplementary Fig. 5 a, Structural comparison of extracellular positively charged residues (Arg and Lys) GPR84 bound to GLPG1205 (purple, receptor in light blue) and ZQ-16 (dark blue, receptor in light pink) with representative free fatty acid receptors, including GPR40/FFAR1-MK8666 (cyan), GPR120/FFAR4-TUG891 (orange), GPR41/FFAR3-valeric acid (yellow), and GPR43/FFAR2-GLPG0974 (light pink). Right panels show electrostatic surface potentials (extracellular view), with blue indicating positive charge and red indicating negative charge. b,c,d, BRET assays of extracellular arginine mutants of GPR84. Alanine substitutions of arginine residues on the extracellular side were evaluated for responses to ZQ-16, 6-OAU, and GLPG1205. e, BRET assay of hydrophobic gate residues mutations of GPR84 in response to 6-OAU. Data are mean ± s.e.m. of $n = 3$ biological replicates. Dose response curves were fitted using nonlinear regression. Statistical significance was assessed using one-way ANOVA.

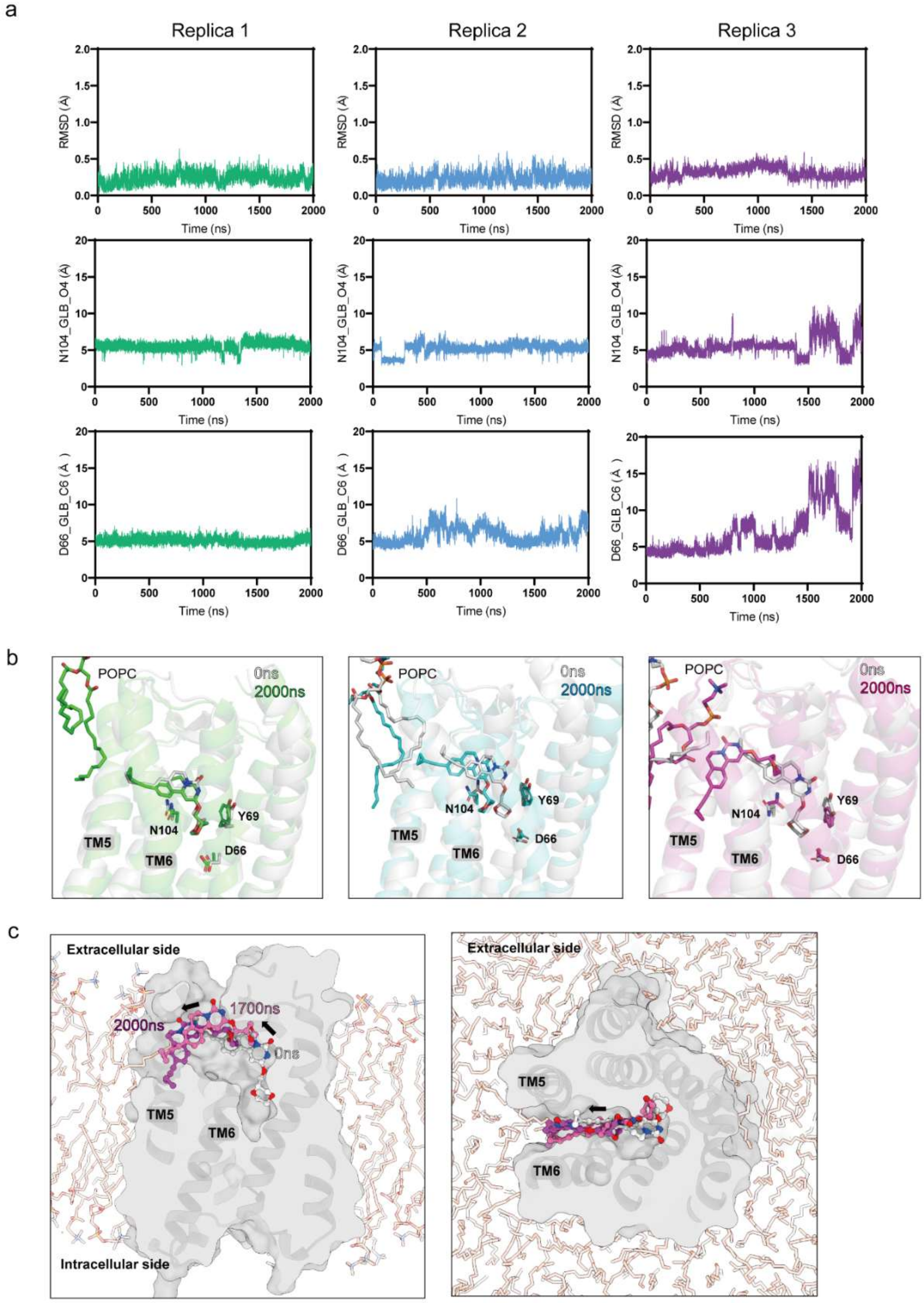

Supplementary Fig. 6 a, Three independent 2 μs MD simulations were performed, and graphs show the analysis of each replica, including ligand RMSD and the time dependent distances between the ligand and key interacting residues. b, The structures at the beginning (0 ns) and end (2000 ns) of each replica were superimposed. Residues critical for ligand interactions and POPC molecules interacting with the ligand between TM5 and TM6 are highlighted. c, Representative snapshots from replica 3 illustrate the ligand migration pathway during the simulation.

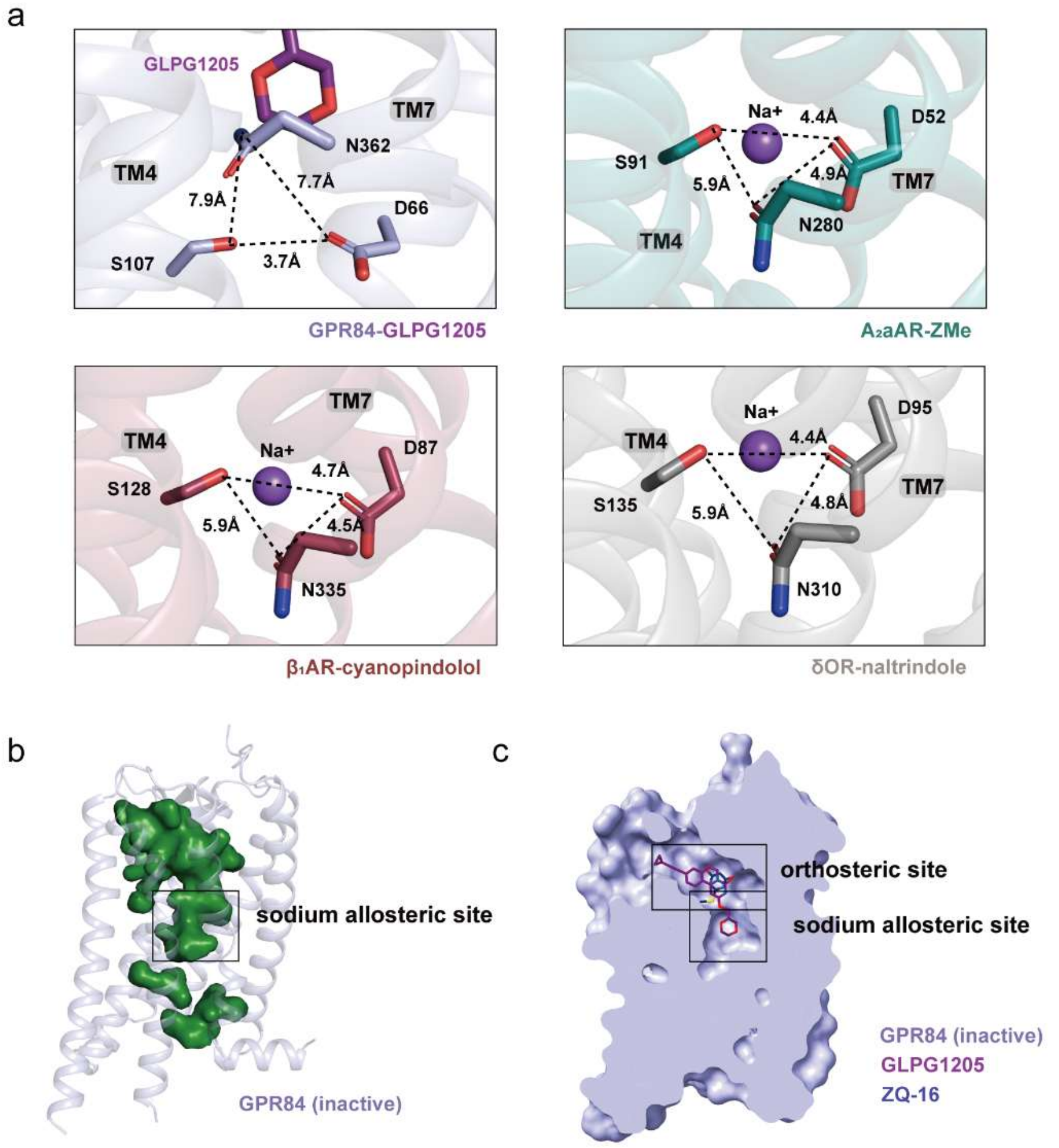

Supplementary Fig. 7 a, Comparison of the sodium binding pocket in GPR84 bound to GLPG1205 (light blue) with canonical sodium coordinated GPCR structures, including A2aAR-ZM241385 (deep teal), β1AR-cyanopindolol (raspberry), and δOR-naltrindole (gray). Distances between coordinating residues are shown (black dashed lines). b, Solvent accessible surface (green) and sodium allosteric site (boxed) in the inactive GLPG1205-GPR84 structure. c, Surface view of the inactive GLPG1205-GPR84 structure highlighting the orthosteric ligand binding site and the sodium allosteric site, with bound GLPG1205 (purple) and ZQ-16 (dark blue).

a

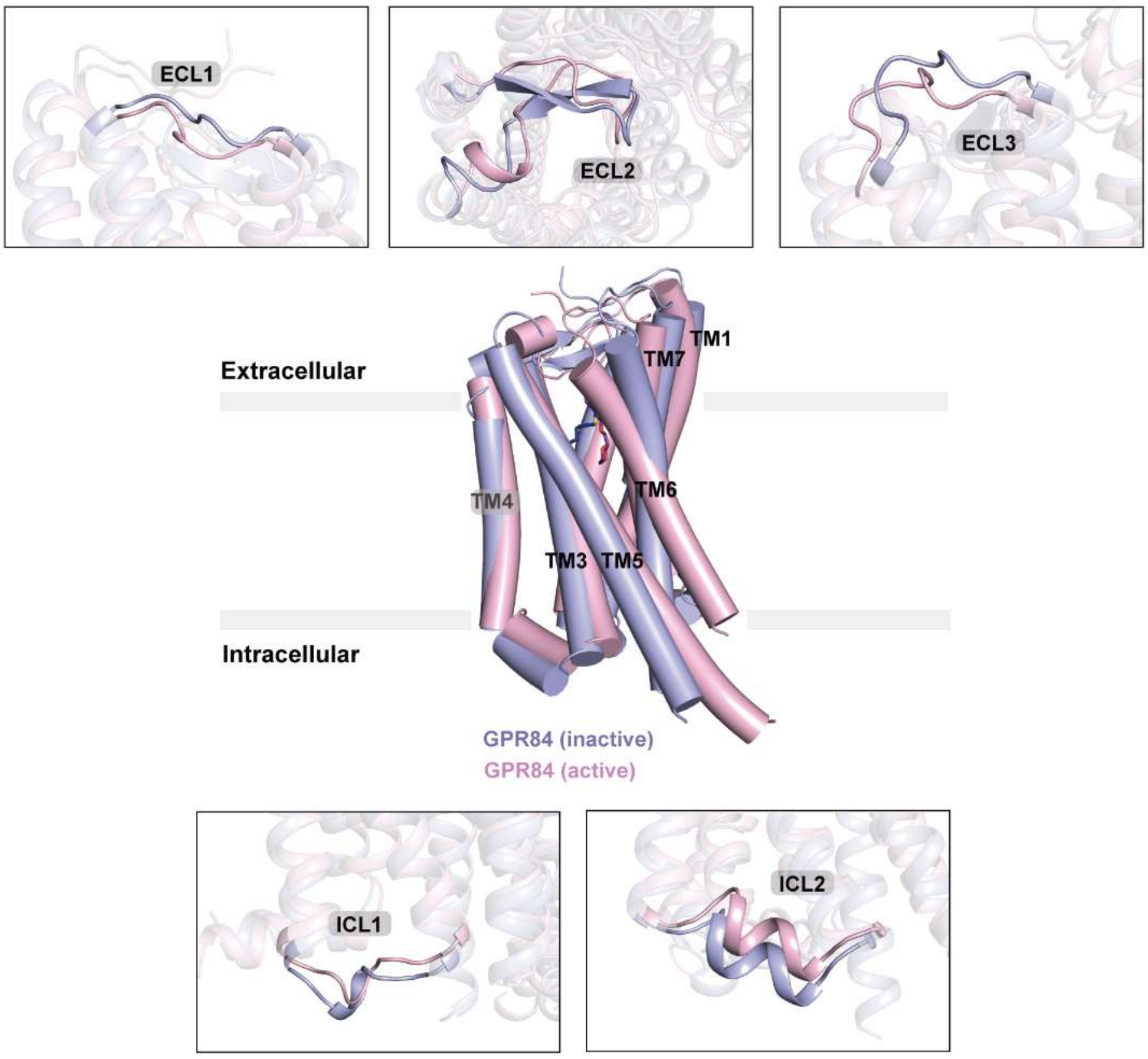

Supplementary Fig. 8 a, Superimposed structures of inactive GLPG1205-GPR84 (light blue) and active ZQ-16-GPR84 (light pink), showing conformational differences in extracellular loops (ECL1-ECL3) and intracellular loops (ICL1-ICL2). The central panel highlights helical movements in the transmembrane domains (TM1-TM7, side view).

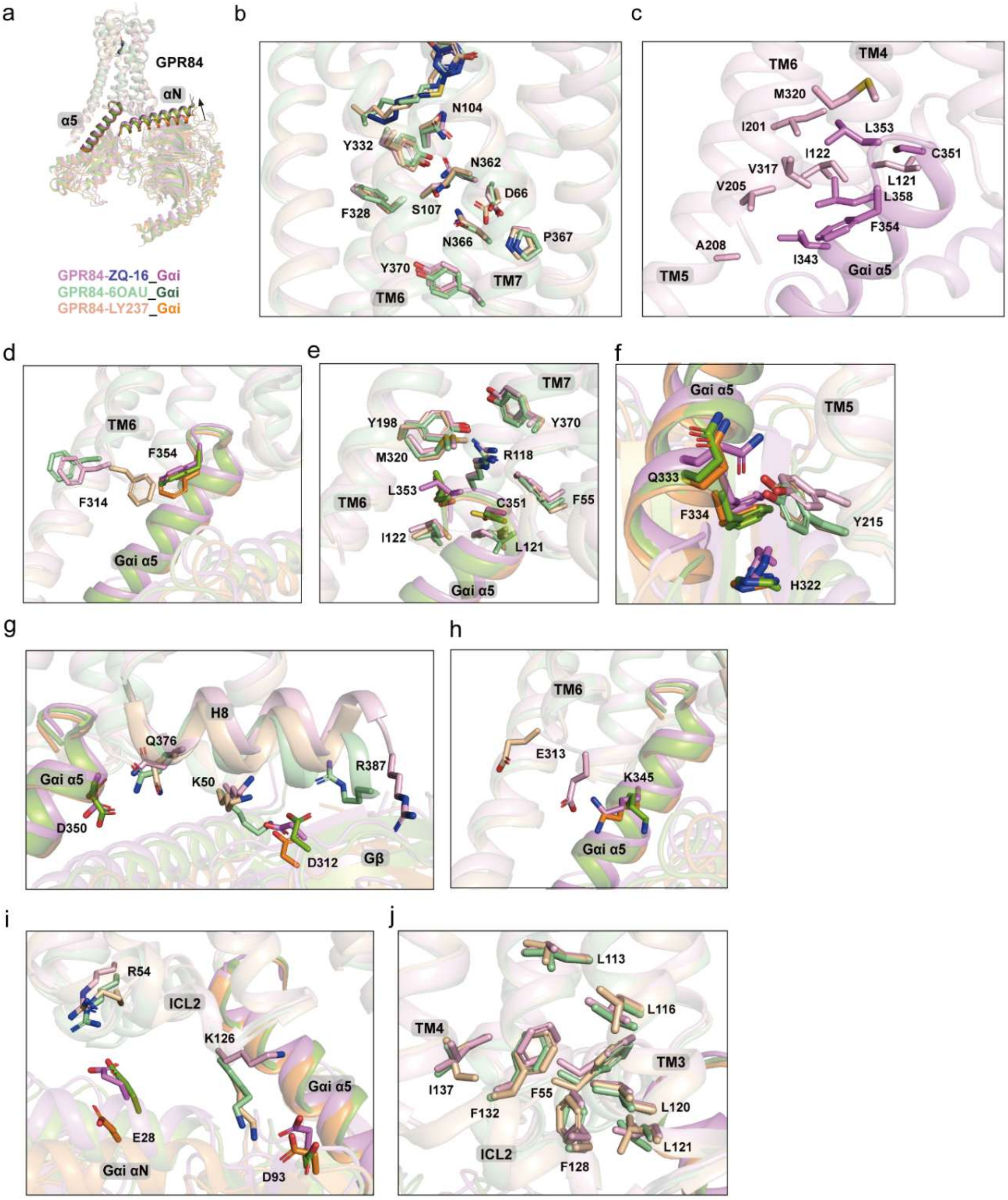

Supplementary Fig. 9 a, Superimposed structures of ZQ-16-GPR84-Gαi (light pink), 6-OAU (light green, PDB: 8G05), and LY237 (light orange, PDB: 8J19) complexes, showing overall alignment of the Gαi α5 helix and αN helix at the receptor interface. b, NPxxY motif. c, Hydrophobic interface between TM4-TM6 of GPR84 and the Gαi α5 helix in the ZQ-16-bound complex. d, TM6- Gαi α5 interface. e, Polar interactions involving TM6 with the Gαi α5. f, Contacts between the Gαi α5, Gβ and helix 8 of GPR84. g, Interactions of Gαi αN and α5 with ICL2 of GPR84. h, TM5- Gαi α5 interface. i, Interface between TM6-TM7 of GPR84 and the Gαi α5 helix. j, Hydrophobic core of ICL2 GPR84.

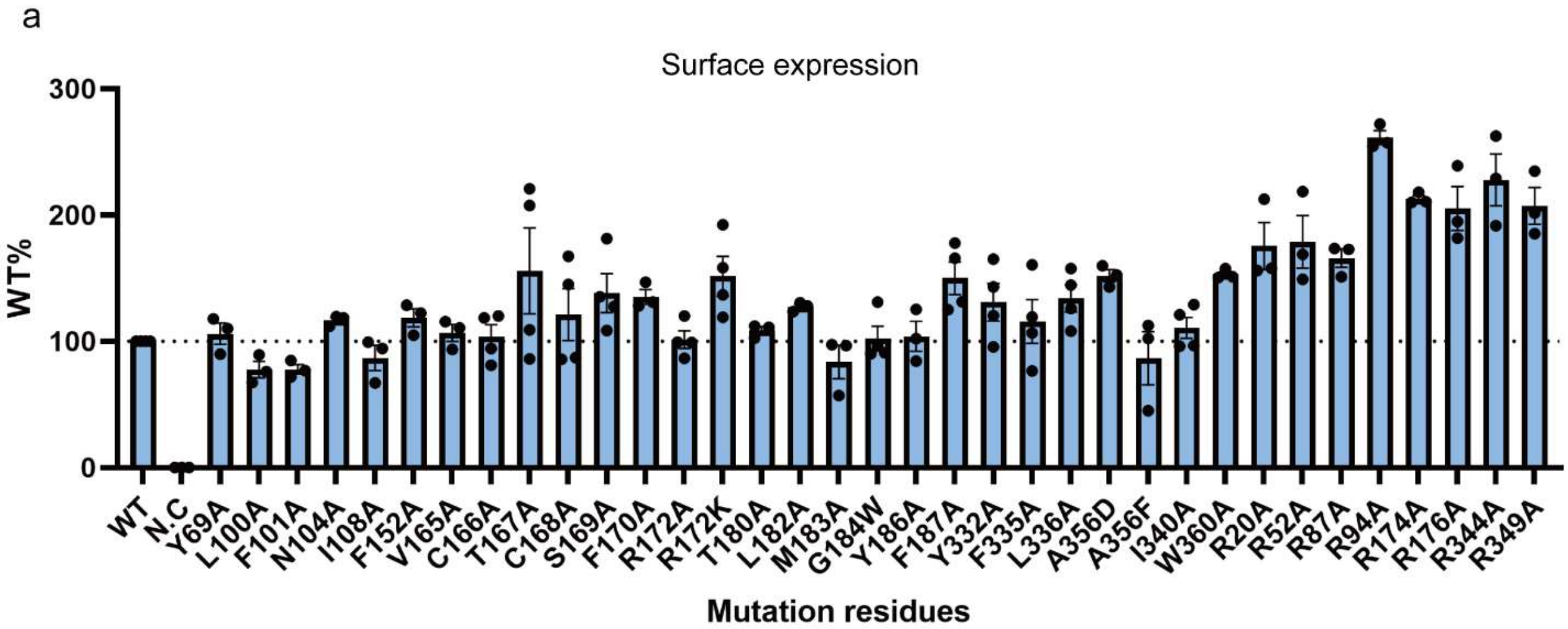

Supplementary Fig. 10 a, ELISA based chemiluminescence quantification of cell surface expression of GPR84 mutants relative to WT. Data are expressed as %WT (mean ± s.d., n = 3).

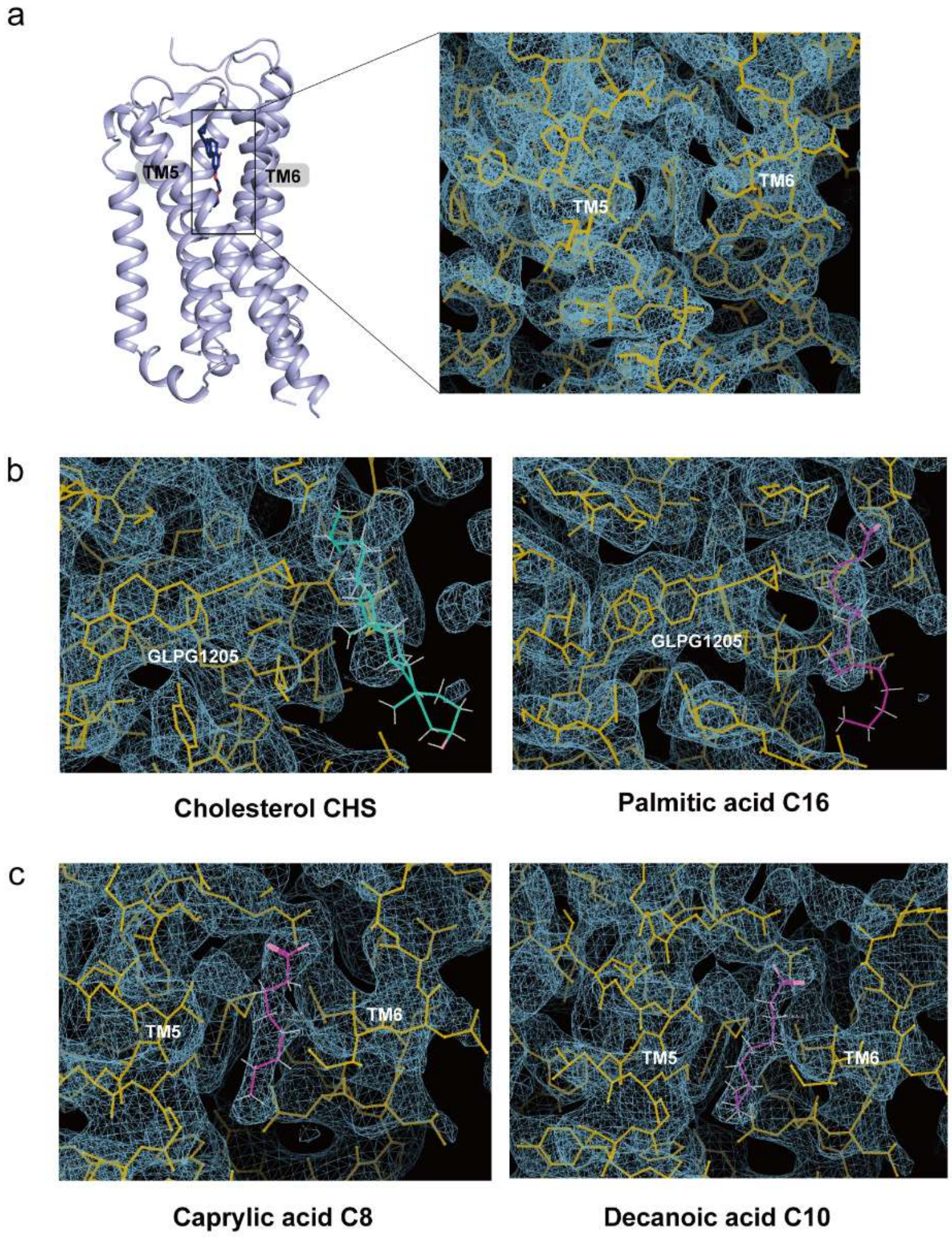

Supplementary Fig. 11 a, GLPG1205-GPR84 structure with a close up of the density between TM5 and TM6, showina lipid-like density feature. b, Cryo-EM densities fitted with cholesterol (CHS, left) and palmitic acid (C16, right) c, Cryo-EM densities fitted with medium chain fatty acids: caprylic acid (C8, left) and decanoic acid (C10, right), shown in stick representation within the density map.

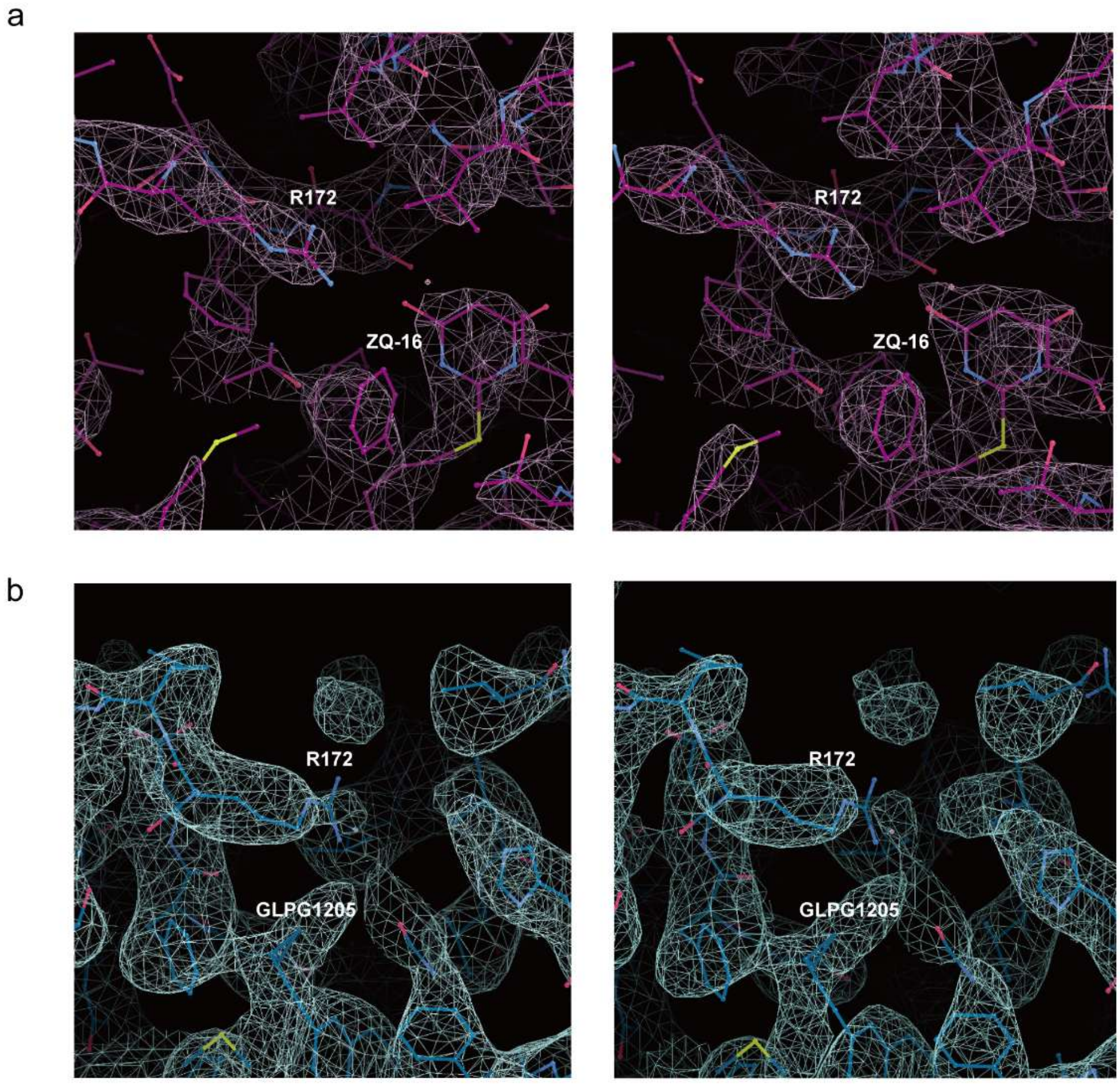

Supplementary Fig. 12 a, Electron density map of Arg172 in the agonist bound structure (ZQ-16), shown as unsharpened (left) and sharpened (right) maps contoured at 12σ. b, Electron density map of Arg172 in the antagonist bound structure (GLPG1205), shown as unsharpened (left) and sharpened (right) maps contoured at 9σ.

a

```
              10         20         30         40         50
GPR84  MWNSSDANFS CYHESVLGYR YVAVSWGVVV AVTGTVGNVL TLLALAIQPK
LY237  MWNSSDANFS CYHESVLGYR YVAVSWGVVV AVTGTVGNVL TLLALAIQPK
6OAU   MWNSSDANFS CYHESVLGYR YVAVSWGVVV AVTGTVGNVL TLLALAIQPK
ZQ-16  MWNSSDANFS CYHESVLGYR YVAVSWGVVV AVTGTVGNVL TLLALAIQPK

              60         70         80         90        100
GPR84  LRTRFNLLIA NLTLADLLYC TLLQPFSVDT YLHLHWRTGA TFCRVFGLLL
LY237  LRTRFNLLIA NLTLADLLYC TLLQPFSVDT YLHLHWRTGA TFCRVFGLLL
6OAU   LRTRFNLLIA NLTLADLLYC TLLQPFSVDT YLHLHWRTGA TFCRVFGLLL
ZQ-16  LRTRFNLLIA NLTLADLLYC TLLQPFSVDT YLHLHWRTGA TFCRVFGLLL

             110        120        130        140        150
GPR84  FASNSVSILT LCLIALGRYL LIAHPKLFPQ VFSAKGIVLA LVSTWVVGVA
LY237  FASNSVSILT LCLIALGRYL LIAHPKLFPQ VFSAKGIVLA LVSTWVVGVA
6OAU   FASNSVSILT LCLIALGRYL LIAHPKLFPQ VFSAKGIVLA LVSTWVVGVA
ZQ-16  FASNSVSILT LCLIALGRYL LIAHPKLFPQ VFSAKGIVLA LVSTWVVGVA

             160        170        180        190        200
GPR84  SFAPLWPIYI LVPVVCTCSF DRIRGRPYTT ILMGIYFVLG LSSVGIFYCL
LY237  SFAPLWPIYI LVPVVCTCSF DRIRGRPYTT ILMGIYFVLG LSSVGIFYCL
6OAU   SFAPLWPIYI LVPVVCTCSF DRIRGRPYTT ILMGIYFVLG LSSVGIFYCL
ZQ-16  SFAPLWPIYI LVPVVCTCSF DRIRGRPYTT ILMGIYFVLG LSSVGIFYCL

             210        220        230        240        250
GPR84  IHRQVKRAAQ ALDQYKLRQA SIHSNHVART DEAMPGRFQE LDSRLASGGP
LY237  IHRQVKRAAQ ALDQYKLRQA SIHSNHVART DEAMPGRFQE LDSRLASGGP
6OAU   IHRQVKRAAQ ALDQYKLRQA SIHSNHVART DEAMPGRFQE LDSRLASGGP
ZQ-16  IHRQVKRAAQ ALDQYKLRQA SIHSNHVART DEAMPGRFQE LDSRLASGGP

             260        270        280        290        300
GPR84  SEGISSEPVS AATTQTLEGD SSEVGDQINS KRAKQMAEKS PPEASAKAQP
LY237  SEGISSEPVS AATTQTLEGD SSEVGDQINS KRAKQMAEKS PPEASAKAQP
6OAU   SEGISSEPVS AATTQTLEGD SSEVGDQINS KRAKQMAEKS PPEASAKAQP
ZQ-16  SEGISSEPVS AATTQTLEGD SSEVGDQINS KRAKQMAEKS PPEASAKAQP
       SEGISSEPVS AATTQTLEGD SSEVGDQINS KRAKQMAEKS PPEASAKAQP

             310        320        330        340        350
GPR84  IKGARRAPDS SSEFGKVTRM CFAVFLCFAL SYIPFLLLNI LDARVQAPRV
LY237  IKGARRAPDS SSEFGKVTRM CFAVFLCFAL SYIPFLLLNI LDARVQAPRV
6OAU   IKGARRAPDS SSEFGKVTRM CFAVFLCFAL SYIPFLLLNI LDARVQAPRV
ZQ-16  IKGARRAPDS SSEFGKVTRM CFAVFLCFAL SYIPFLLLNI LDARVQAPRV

             360        370        380        390
GPR84  VHMLAANLTW LNGCINPVLY AAMNRQFRQA YGSILKRGPR SFHRLH
LY237  VHMLAANLTW LNGCINPVLY AAMNRQFRQA YGSILKRGPR SFHRLH
6OAU   VHMLAANLTW LNGCINPVLY AAMNRQFRQA YGSILKRGPR SFHRLH
ZQ-16  VHMLAANLTW LNGCINPVLY AAMNRQFRQA YGSILKRGPR SFHRLH
```

Supplementary Fig. 13 a, Secondary structure alignment of human GPR84 with representative ligand bound complexes, including LY237, 6-OAU, and ZQ-16. Residues are numbered according to the GPR84 sequence. Secondary structure elements are color coded as: gray, disordered region; green, loop; blue, α helix; purple, β sheet.

Supplementary Table 1: Cryo-EM data collection and refinement statistics

	GLPG1205-bound GPR84 complex	ZQ16-bound GPR84-Gi complex
EMDB	64807	64407
PDB ID	9V6T	9UPY
**EM data collection**		
Microscope	Titan Krios G4	Titan Krios G4
Detector	Gatan K3 BioQuantum	FEI FALCON IV
Magnification	130,000	160,000
Volatage (kV)	300	300
Defocus range (μm)	-0.7 ~ -2.0	-0.8 ~ -2.1
Electron dose (e-/Å2)	69	50
Pixel size (Å)	0.664	0.7052
Number of movies	8,575	16,820
**Data processing**		
Number of extracted particles	2,680,402	3,535,353
Number of particles for final map	134,250	204,250
Symmetry imposed	C1	C1
Resolution (Å)	3.5	3.17
FSC threshold	0.143	0.143
**Refinement**		
Initial model used	AlphaFold 2 + PDB: 6WW2	AlphaFold 2 + PDB: 8G05, 8J19
Composition (#)		
Atoms	4,855	7,000
Amino acids	4,827	6,969
Ligands	28	31
RMSD bonds (Å) (# > 4σ)	0.005	0.004
RMSD angles (°) (# > 4σ)	0.714	0.564
Mean B-factors (Å2)		
Amino acids	88.01	128.09
Ligand	95.31	93.87
Ramachandran (%)		
Favored	93	96
Allowed	6	4
Outliers	0	0
Rotamer outliers (%)	0	0
Clash score	9.58	22
C-beta outliers (%)	0	0
CaBLAM outliers	2.5	1.27
CC (mask)	0.78	0.81
MolProbity score	1.92	2.14

Supplementary Table 2: Summary of BRET assay parameters for GPR84 mutants

Ligand	Construct	EC50 (nM)	Emax	Residual Activity
ZQ-16	WT	640	100.0	
	R20A	195	19.5	Loss of function
	R52A	111	29.2	Loss of function
	Y69A	1181	72.6	Partial loss
	R87A	6.84	20.5	Loss of function
	R94A	1644	27.2	Loss of function
	L100A	36700	35.0	Loss of function
	F101A	367	66.4	Partial loss
	N104A	0.65	6.7	Inactive
	I108A	855	18.3	Loss of function
	F152A	32.79	99.7	Increased potency
	V165A	24480	97.6	Reduced potency
	T167A	4029	49.5	Partial loss
	S169A	46.95	14.7	Inactive
	F170A	1495	30.1	Loss of function
	R172A	25.38	6.5	Inactive
	R172K	3431	30.8	Loss of function
	R174A	961	97.5	WT like
	R176A	6686	87.3	Reduced potency
	T180A	763	100.1	WT like
	L182A	846	85.6	Reduced potency
	M183A	12260	77.6	Reduced potency
	G184W	1939	91.3	Reduced potency
	Y186A	1536	83.3	Reduced potency
	F187A	297	108.8	Increased potency
	Y332A	8.04	5.0	Inactive
	F335A	831	23.0	Loss of function
	L336A	1467	47.4	Partial loss
	I340A	38050	61.8	Partial loss
	R344A	1278	97.6	WT like
	R349A	6530	30.0	Loss of function
	A356F	89.23	0.7	Inactive
	A356D	0.31	-6.3	Inactive
	T359A	1955	51.6	Partial loss
	W360A	593	34.2	Loss of function
6OAU	WT	1820	100.0	
	R20A	0.62	3.4	Inactive
	R52A	0.24	6.0	Inactive
	R87A	10110	26.5	Loss of function
	R94A	7397	48.2	Loss of function
	R172A	ND	ND	Inactive
	R174A	2239	112.3	WT like
	R176A	8535	35.2	Loss of function
	T180A	2591	102.8	WT like
	G184W	5937	70.4	Partial loss
	L336A	5639	60.4	Partial loss
	I340A	13590	40.0	Partial loss
	R344A	3997	90.9	WT like
	R349A	664	52.0	Partial loss

Ligand	Construct	IC50 (nM)	Emax	Residual Activity
GLPG1205	WT	207.7	0.0	
	R20A	47.27	92.7	Loss of function
	R52A	5.08	98.1	Loss of function
	Y69A	15500	51.2	Loss of function
	R87A	1.54	111.3	Loss of function
	R94A	86.55	67.4	Loss of function
	L100A	0.51	103.5	Loss of function
	F101A	7285	27.9	Partial loss
	N104A	1.18	87.7	Loss of function
	V165A	6.15	85.3	Loss of function
	C166A	0.07	76.9	Partial loss
	C168A	65.71	83.3	Loss of function
	F170A	1362	75.1	Partial loss
	R172A	23.62	101.1	Loss of function
	R172K	5.78	93.9	Loss of function
	R174A	151	4.3	WT like
	R176A	6.9	84.6	Loss of function
	T180A	141	95.6	Loss of function
	M183A	ND	ND	Inactive
	G184W	24.92	96.8	Loss of function
	F187A	555	86.5	Loss of function
	F335A	239	114.3	Loss of function
	L336A	36.38	93.1	Loss of function
	I340A	4.03	101.2	Loss of function
	R344A	51.05	21.5	Partial loss
	R349A	251	60.5	Loss of function
	A356F	82.47	102.5	Loss of function
	A356D	ND	ND	Inactive
	W360A	ND	ND	Inactive

WT like, WT E_{max} 80-120% with no significant change in EC_{50}/IC_{50}; **Reduced potency**, WT E_{max} 80-120% with 3-5 fold increase in EC_{50}/IC_{50}; **Increased potency**, WT E_{max} 80-120% with 3-5 fold decrease in EC_{50}/IC_{50}; **Partial loss**, WT E_{max} 50-80%; **Loss of function**, WT E_{max} 10-50%; **Inactive,** WT E_{max} <10% or not determined (ND).
